# T-Cell Epitope Prediction: Rescaling Can Mask Biological Variation between MHC Molecules

**DOI:** 10.1371/journal.pcbi.1000327

**Published:** 2009-03-20

**Authors:** Aidan MacNamara, Ulrich Kadolsky, Charles R. M. Bangham, Becca Asquith

**Affiliations:** Department of Immunology, Imperial College School of Medicine, London, United Kingdom; Utrecht University, The Netherlands

## Abstract

Theoretical methods for predicting CD8+ T-cell epitopes are an important tool in vaccine design and for enhancing our understanding of the cellular immune system. The most popular methods currently available produce binding affinity predictions across a range of MHC molecules. In comparing results between these MHC molecules, it is common practice to apply a normalization procedure known as rescaling, to correct for possible discrepancies between the allelic predictors. Using two of the most popular prediction software packages, NetCTL and NetMHC, we tested the hypothesis that rescaling removes genuine biological variation from the predicted affinities when comparing predictions across a number of MHC molecules. We found that removing the condition of rescaling improved the prediction software's performance both qualitatively, in terms of ranking epitopes, and quantitatively, in the accuracy of their binding affinity predictions. We suggest that there is biologically significant variation among class 1 MHC molecules and find that retention of this variation leads to significantly more accurate epitope prediction.

## Author Summary

The use of prediction software has become an important tool in increasing our knowledge of infectious disease. It allows us to predict the interaction of molecules involved in an immune response, thereby significantly shortening the lengthy process of experimental elucidation. A high proportion of this software has focused on the response of the immune system against pathogenic viruses. This approach has produced positive results towards vaccine design, results that would be delayed or unobtainable using a traditional experimental approach. The current challenge in immunological prediction software is to predict interacting molecules to a high degree of accuracy. To this end, we have analysed the best software currently available at predicting the interaction between a viral peptide and the MHC class I molecule, an interaction that is vital in the body's defence against viral infection. We have improved the accuracy of this software by challenging the assumption that different MHC class I molecules will bind to the same number of viral peptides. Our method shows a significant improvement in correctly predicting which viral peptides bind to MHC class I molecules.

## Introduction

Cytotoxic T lymphocytes (CTLs) discriminate between healthy and pathogen-infected cells by recognizing and responding to a molecular complex on the surface of the infected cell. This complex consists of a specific major histocompatibility complex (MHC) molecule and a peptide derived from the proteins contained in the cell. If the cell contains a pathogen, peptides from the pathogen proteome will be presented and, with the right MHC – peptide complex, a CTL response will be elicited.

Of the large number of peptides that can be derived from a pathogen only a small minority elicits a CTL response. This number has been estimated to be between 1 in 2,000 and 1 in 5,600 [1],[2]. This limitation in the number of peptides that are immunogenic is conferred by three main constraints: the requirement for peptide cleavage and transport, the requirement for MHC-peptide binding and the requirement for CTL recognition. By far the most stringent of these is the requirement for MHC-peptide binding, because only 1 in 40–200 peptides binds a specific MHC molecule with sufficient affinity to elicit an immune response [1],[2]. Further selection is largely due to the limitations of peptide processing and transport. In these processes, individual peptides are produced from the precursor polypeptides by proteasomal cleavage of the polypeptide, which can be followed by N-terminal trimming by other peptidases. This is followed by the transport of the peptides from the cytosol to the endoplasmic reticulum, mediated by the TAP complex. Further N-terminal trimming may occur before the peptide binds to the MHC molecule. The requirements of processing and transport eliminate approximately 80% of potential epitopes [1]. Finally, T cell specificity, i.e. the requirement for T cell receptor binding of the MHC-peptide complex, further halves the number of presented peptides that elicit a response. The probability of each of these steps is determined by the polypeptide sequence, amongst other factors [3].

Once CTLs recognize the MHC-peptide complex, they are capable of destroying the infected cell by the release of lytic granules containing cytotoxic effector proteins. This results in the destruction of the target cell by apoptosis. An effective CTL response has been shown to confer protection against viral infection, such as HIV [4] and HTLV-I [5]. Hence, the identification of T cell epitopes is of vital importance in the design of vaccines and understanding of the immune system [6],[7],[8]. However, given the scarcity of epitopes, experimentally screening all possible peptides for each MHC allele (e.g. by IFNγ ELISpot) is time consuming, expensive and inefficient. One way to improve the efficiency of the identification process is to first use theoretical algorithms to predict which peptides are more likely to be epitopes and then experimentally screen this much smaller, selected list of peptides. This method is widely used [9]–[12] and has been applied in a number of studies to identify potential vaccine targets [13],[14]. The use of theoretical methods to “pre-screen” peptides is of particular importance in the case of emerging infections such as avian influenza [15] where rapid vaccine development would be vital. This approach underpins a large bio-preparedness initiative coordinated by the Large-Scale Antibody and T Cell Epitope Discovery Program [7], which intends to foster development of immune-based therapeutics for emerging and reemerging pathogens including potential bioterrorism agents. The accuracy of these methods has also been demonstrated by the prediction of the vast majority of CTL epitopes from the vaccinia virus [16].

More generally, epitope prediction algorithms are being increasingly used to understand the CTL response. For example, in the case of HIV-1 infection, algorithms have been used to confirm which MHC-associated epitope mutations are likely to confer escape from a CTL response [17] and to understand why some MHC class I alleles are associated with slow rates of disease progression [18],[19].

A range of computational algorithms have been developed to predict CTL epitopes in pathogen protein sequences. Since the most selective requirement for a peptide to be immunogenic is the ability of the peptide to bind to the MHC molecule, most prediction methods focus on this stage of the pathway. As a general rule, information gained from experimental binding assays is used to train the algorithm until it is efficient at predicting novel MHC–peptide complexes. The algorithms that are used vary in complexity and accuracy. Some can be trained to recognize peptide motifs that are required for binding to a particular MHC molecule [20], others use a weight-matrix method to identify amino acids that occur at a higher-than-expected frequency at specific epitope positions [21],[22],[23]. However, the most accurate methods available use logistic regression [24] and, more generally, artificial neural networks [3].

Artificial neural networks (ANNs) take into account, in addition to the identity of each amino acid residue, the interactions between adjacent amino acids in a potential epitope. In summary, an ANN for a particular MHC molecule is trained to recognize associated inputs (a peptide sequence) and outputs (the binding affinity for that sequence with the MHC molecule) [25]. Once an ANN is trained for a particular molecule, it can predict the binding affinity of novel peptide sequences.

NetCTL [3] and NetMHC [25],[26],[21] are two of the most accurate prediction methods currently available [27]. NetMHC uses ANNs for a number of alleles to predict MHC molecule-peptide binding affinities. NetCTL, as well as using ANNs to predict MHC – peptide binding, also utilizes information about the proteasomal cleavage of the input peptide sequence, and its ability to bind to TAP. NetCTL or NetMHC will predict a score (either integrated or simply a binding affinity, respectively) for every overlapping nonamer peptide sequence in an input sequence to each MHC molecule for which the method has an ANN. Henceforth, we refer to the trained prediction algorithm for each MHC class I allele as an “allelic predictor”.

### Rescaling

In order to make the prediction values comparable between each MHC molecule, it is recommended that the MHC-peptide binding affinity scores are rescaled [28]; this is explicitly implemented in NetCTL. The method of rescaling involves obtaining the predicted binding affinities of 500,000 random natural peptides for each MHC allelic predictor. From these affinities, a rescale value is calculated, defined as the binding affinity that is the threshold for the top 1% of total binding affinities. The rescaled affinity is then defined as the predicted affinity score divided by this rescale value [3]. Hence, from this calculation, all alleles are predicted to bind the same number of high-affinity peptides. One pragmatic reason for rescaling is to correct for any discrepancies between the allelic predictors that resulted from inconsistent training data (e.g. data that came from different sources), by assuming that all alleles should bind the same number of epitopes (C. Keşmir, pers. comm.). Additionally, there are biological arguments for believing that different alleles should bind similar numbers of epitopes. It has been postulated that the opposing constraints of effective pathogen recognition but tolerance of self would result in a very narrow range of optimal promiscuity for viable MHC class I molecules. A narrow range of promiscuity would also be predicted as a direct outcome of effective tapasin-dependent peptide optimization in the endoplasmic reticulum [29],[30],[31].

However, we will present evidence in this paper that in correcting for differences between the allelic predictors, information is being lost that reflects true biological variation between MHC molecules and, by extension, differences in their ability to bind to peptide sequences. We show that, for both qualitative and quantitative measures of binding, rescaling impairs rather than improves allelic predictor performance. This is of importance for vaccine design and to understand the nature of the CTL response. In particular, crucial between-allele variations in binding affinity and preference which may contribute to differences in the outcome of infection are likely to be obscured by rescaling.

## Methods

### Prediction Method Outputs

In order to test the effect of rescaling on epitope prediction accuracy, we used two web-based prediction methods, NetCTL v1.2 [3] and NetMHC v3.0 [25],[26],[21]. NetCTL is an integrated method that uses information pertaining to TAP and protein cleavage in its predictions, together with MHC binding. The output is combined by rescaling the MHC binding result and adding this to the weighted scores for TAP and protein cleavage. NetCTL has allelic predictors for 12 different class I alleles that are chosen to be representative of each of 12 supertypes; hence it has 12 different rescaling factors.

NetMHC v3.0 simply predicts MHC-peptide binding, using ANNs to predict binding affinities for 43 MHC molecules. In order to test the effect of rescaling, it was necessary to produce rescale values for each of the 43 allelic predictors. This was performed as in NetCTL; 500,000 unique random nonamers were obtained from the proteome of *Mycobacterium tuberculosis*, their binding affinity was predicted and the rescale value (top percentile) was found for each allelic predictor. We also performed this calculation with 500,000 random natural peptides to test for the possibility of error from bias in amino acid usage in *Mycobacterium tuberculosis*. There was no significant difference in the rescale values obtained using these two different sources (supplementary material, figure S4).

In summary, we tested two sets of rescaling values: those obtained from NetCTL v1.2 and those that we calculated using NetMHC v3.0.

### Datasets

Epitope datasets were constructed from sources detailed below. In each case, the prediction methods were tested by their ability to detect these epitopes amongst the full set of overlapping nonamers derived from the proteins that contained the epitopes. The full set of nonamers will contain a small number of known epitopes and the remainder will be ‘non-epitopes’. Of course, this set of non-epitopes could include epitopes that have not been experimentally verified. However, the majority (see Introduction) would be non-binders with the corresponding MHC molecule. Added to this, the labelling of epitopes as ‘non-epitopes’ impact on both rescaled and non-rescaled calculations equally. Previous research has also shown that this property of the ‘non-epitope’ set did not produce significantly different results [24]. Each respective set of experimentally defined epitopes was denoted the positive dataset and the set of non-binding (or unknown) peptides was denoted the negative dataset.

### The SYF^1^ Dataset

The SYF^1^ dataset is a supertype dataset derived from SYFPEITHI [20] and is identical to that used in the original paper for NetCTL [3]. Each epitope in SYF^1^ was experimentally verified to bind to one of 10 MHC class I supertypes [32]. The resulting dataset consisted of 148 epitope-supertype pairs. The corresponding negative dataset was obtained by concatenating the SwissProt entry proteins from which each of the epitopes was derived. The length of the concatenated protein sequence was 78,259 amino acids. The ROC curve (see below for explanation) was generated using a negative set of ((78,259*10)−148) = 782,442 nonamers and a positive set of 148 nonamers. The positive set of SYF^1^ is available in the supplementary material (dataset S2).

### The Lanl^661^ Dataset

Experimentally defined epitopes in HIV-1 were extracted from the HIV Molecular Immunology Database [33]. In total, 1,618 CTL epitopes were found that were bound by human MHC molecules. However, this set was highly redundant; the epitope lengths were variable and a large number of epitopes differed only by mutations within the sequence. Also, resolution of their MHC typing varied from 2 to 4 digits. To correct for this variability, a number of changes were made to the MHC allele-epitope list. Firstly, all MHC alleles were defined to two digits. Secondly, variant epitopes binding the same allele were discarded. Finally, as the prediction software only produced binding predictions for nonamer epitopes, all epitopes that were not 9 amino acids long were removed from the list.

In summary, it was possible to test 41 of the 43 allelic predictors for MHC molecules in NetMHC v3.0. The positive set consisted of 661 epitopes, defined in terms of start and end positions relative to the HIV reference strain HXB2 (supplementary dataset S1) and a matching MHC type to 2 digits. The input protein sequence to NetMHC contained 3,000 overlapping nonamers that covered the proteome from which the whole positive set of epitopes was derived. The total ‘negative set’ for the ROC analysis was (3,000 * 41)−661) = 122,339 nonamers, and a positive set of 661 nonamers. The positive set of Lanl^661^ is available in the supplementary material (dataset S3).

### The Lanl^179^ Dataset

The Lanl^661^ dataset was modified for testing with NetCTL. From these 661 epitopes, a total of 179 bound to the 12 alleles for which NetCTL has allelic predictors. The input sequence to NetCTL contained 3,000 overlapping nonamers. For this experiment, the negative set consisted of ((3,000 * 12)−179) 35,821 nonamers, and a positive set of 179 nonamers. The positive set of Lanl^179^ is available in the supplementary material (dataset S4).

### ROC Curves

ROC curves give a visual measure of the accuracy of a prediction method. The threshold at which the prediction method identifies a peptide as being an epitope varies along the length of the curve. Each point on the curve gives the fraction of true positive epitopes found as a function of the number of false positive ‘epitopes’ at that threshold. Hence, setting a strict threshold for epitope detection will result in high specificity (correct predictions) but low sensitivity (missing a high proportion of true binders). The area under the ROC curve gives the AUC (Area under Curve) measurement. In order to test for significant difference between ROC curves, we conducted the bootstrapping analysis detailed in [34]. Briefly, using bootstrapping with replacement, 100 replicates were formed from each dataset and the resulting non-rescaled and rescaled whole AUC values were compared using a paired *t* test.

### Other Measurements of Performance

Using the 2 epitope datasets, HIV^216^ and SYFPEITHI^863^, and the same methods from [35], we repeated 3 of the measurements described in that paper for the rescaled and non-rescaled results of NetCTL v1.2. For the Rank measure, we analysed the proteins from which each epitope was derived. For each protein, we calculated the rank of the epitope amongst all overlapping 9-mers using rescaling and non-rescaling scoring methods for all alleles. We then analysed these ranks to see which method ranked the epitopes higher. For the second method, we measured the specificity of both rescaling and non-rescaling at predefined sensitivities. Finally, we measured the sensitivity among the top 5% top-scoring peptides, again for the rescaled and non-rescaled binding affinities.

### Other Data Sources

The training data for NetMHC v3.0 is available at http://mhcbindingpredictions.immuneepitope.org/. An independent set of experimental epitope-allele binding affinities was obtained from the Immune Epitope Database and Analysis Resource (IEDB) by selecting all experimental data that did not originate from the laboratories of Sette *et al.* or Buus *et al.* (the training data originated from these two sources).

## Results

### The Effect of Rescaling on Qualitative Epitope Prediction

**Figure 1:**
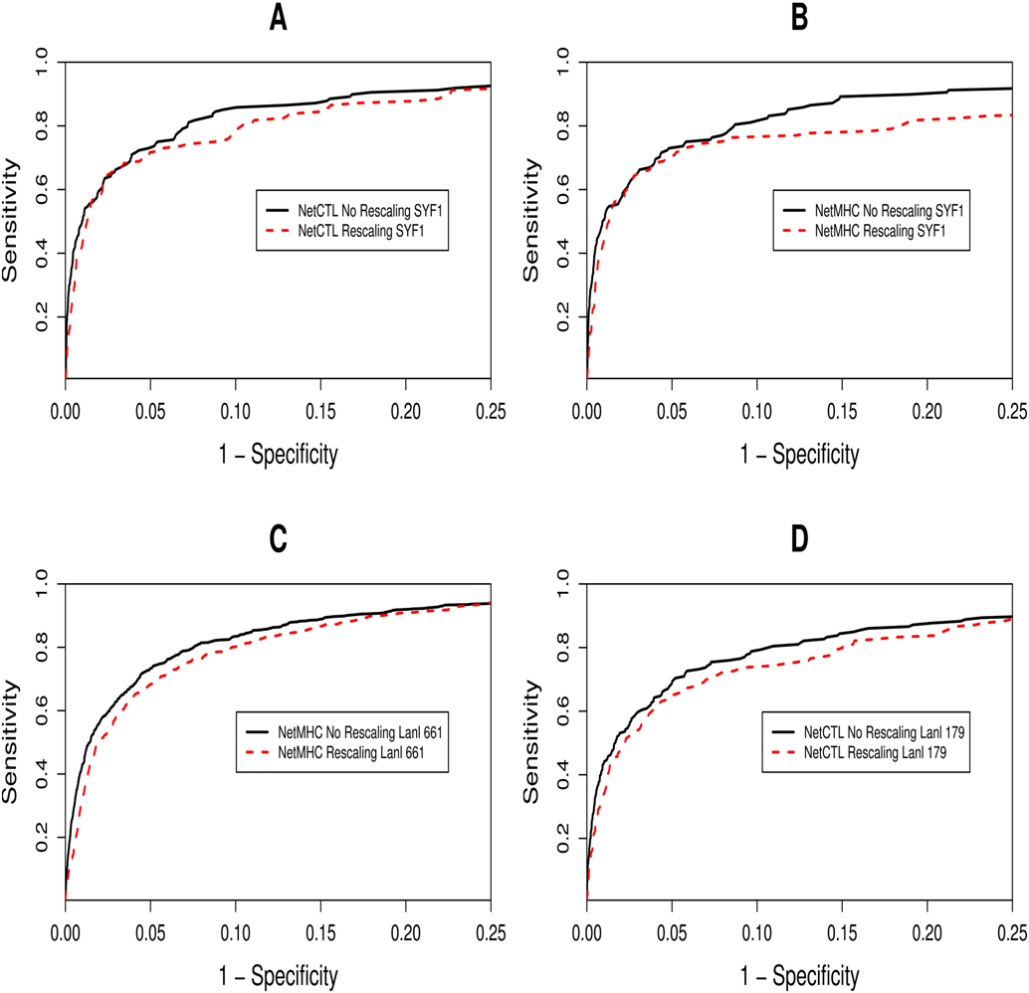
ROC curve analysis on the effects of rescaling. Each graph shows the ROC curves using different combinations of datasets and prediction methods (see table 1). Figure 1A uses NetCTL with the SYF^1^ dataset, figure 1B NetMHC with the SYF^1^ dataset, figure 1C NetMHC with the Lanl^661^ dataset and figure 1D NetCTL with the Lanl^179^ dataset. The x-axis has been scaled to show the region of importance (the AUC with high specificity values). The rescaled results (red dashed line) are compared against non-rescaled (black solid line). Table 1 gives the statistics for each graph.

ROC curves were used to analyse the effects of rescaling on epitope prediction. Both NetCTL v1.2 and NetMHC v3.0 were tested and 3 datasets were used (figure 1 and table 1). In each case, rescaling resulted in a significant loss of performance (bootstrap test: p<0.001).

**Table 1:** The summary statistics and details of each ROC curve from figure 1.

ROC Curve	Colour	Method	Dataset	Rescaling	AUC	Bootstrap P-Value
Figure 1A	Black solid	NetCTL v1.2	SYF^1^	No^a^	0.949	<0.001
	Red dashed	NetCTL v1.2	SYF^1^	Yes	0.937	
Figure 1B	Black solid	NetMHC v3.0	SYF^1^	No	0.932	<0.001
	Red dashed	NetMHC v3.0	SYF^1^	Yes	0.905	
Figure 1C	Black solid	NetMHC v3.0	Lanl^661^	No	0.944	<0.001
	Red dashed	NetMHC v3.0	Lanl^661^	Yes	0.937	
Figure 1D	Black solid	NetCTL v1.2	Lanl^179^	No^a^	0.933	<0.001
	Red dashed	NetCTL v1.2	Lanl^179^	Yes	0.918	

a In NetCTL v1.2, the TAP and cleavage scores are combined with the rescaled MHC binding score to produce a combined score for each submitted nonamer. In order to test how NetCTL performed without rescaling, it was still necessary to divide the MHC binding score by a rescaling value so the weightings of the TAP and cleavage score were still applicable and accurate. By averaging over all rescaling values and dividing the MHC binding value by this number, rescaling differences were “averaged out” and it was still possible to use the extra information from the TAP and cleavage predictions.

### Variation in Rescale Values as a Function of Accuracy

**Figure 2:**
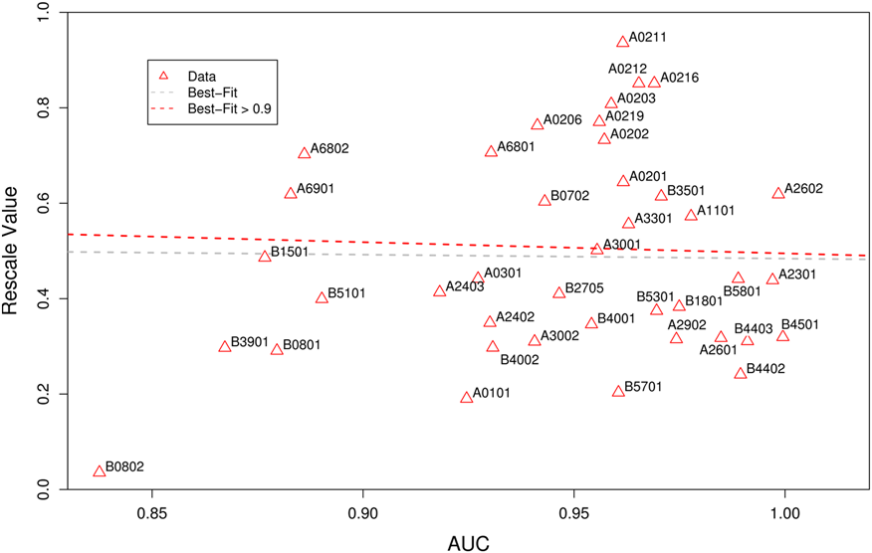
The relationship between AUC and rescale value. There is no evidence for a correlation of AUC and rescale value for the whole set of allele predictors (R^2^ = 0.0068, p = 0.606), nor for the subset of predictors with an AUC>0.9 (R^2^ = 0.0007, p = 0.887). This analysis used the Lanl^661^ epitope dataset.

One possible explanation for why rescaling has a detrimental impact on prediction is that there may be a positive correlation between rescale factor and allelic predictor accuracy. To check this hypothesis we calculated the AUCs for each NetMHC v3.0 predictor using the Lanl^661^ dataset and plotted this against the corresponding rescale factor, the results of which are shown in figure 2. This shows no evidence of a correlation between rescaling values and the AUC values (R^2^ = 0.0068, p = 0.606).

Consequently, it is unlikely that a correlation between rescale values and AUC values explains our findings. However, certain alleles like B0801 do have both a low rescale value and a low AUC. To double check that these poor accuracy predictors were not causing the inaccuracies in rescaled predictions we repeated our ROC curve analysis for Lanl^66^1 without the low accuracy predictors (those with an AUC value below 0.9; namely A6801, A6802, B3501, B0702, B0801, B0802 and B4501). In the remaining, reduced subset of predictors there was even less evidence for a correlation between AUC and rescale factor (R^2^ = 0.0007, p = 0.887). For this subset of predictors the accuracy was still significantly better if rescaling was not applied (figure S1; bootstrap test: p<0.001) and comparable to the ROC curve analysis using the full set of alleles (figure 1C).

Therefore, we believe there is no evidence to support the hypothesis that the reason rescaling is detrimental is because there is a correlation between rescale factors and AUC.

### Other Measurements of Performance

We used 3 other metrics [35] to compare predictive performance with and without rescaling.

1. The rank of known epitopes was compared with non-epitopes from the same protein for both rescaled and non rescaled predictions. From figure S2, it can be seen that the non-rescaled results produced significantly more accurate results for both epitope datasets (paired Wilcoxon ranked sum test, P<0.001).
2. Non-rescaling predicted binding affinities produced improved results compared to rescaling at given sensitivities using the epitope datasets from [35] (supplementary table S1).
3. Non-rescaling predicted binding affinities also produced improved results comparing the total number of epitopes among the top 5% predicted binding affinities (supplementary table S2), again using the epitope datasets from [35].

### The Effect of Rescaling on Quantitative Predictions of Binding Affinities

Using 2 sets of experimentally-derived epitope-allele binding affinities, we also showed that the correlation between predicted and experimental affinities was weaker with rescaling than without (supplementary figure S3).

## Discussion

Rescaling is, in theory, a sound approach to improving epitope prediction and in particular comparability of predictions obtained using different allelic predictors. However, using a number of different measures of accuracy, in the context of two commonly used prediction methods, we have demonstrated that rescaling actually impairs rather than improves predictive performance and comparability. We suggest that rescaling predicted affinities results in a loss of information that outweighs any advantage gained in correcting for differences in training data.

The first approach used ROC curve analysis and showed clear differences between rescaling and non-rescaling. The ROC curve gives a graphical representation of how well the prediction method ranks true epitopes among a set of non-binding peptides. Or to use an analogy, how efficient it is at finding the epitopic needle in a haystack of random peptides. From figure 1, it is clear that rescaling across all allelic predictors results in a performance loss in terms of how well the method ranks its peptides by binding affinity; that is, rescaling impairs intra-allelic comparisons. This loss could be demonstrated using epitope data from a number of sources (SYFPEITHI, the HIV Molecular Immunology Database) and with two different methods of prediction (the combined approach of NetCTL v1.2 and NetMHC v3.0). This effect of rescaling would be detrimental to any studies screening across a number of alleles for possible epitopes (such as [15]). The effect of this performance difference can be gauged from figure 1 (A). In order to identify correctly 85% of the epitopes the percentage of false positives detected was 9% and 15%, for non-rescaled and rescaled methods respectively. To put this result into context, the viral protein NS1 from the H5N1 strain of Avian Influenza A consists of 221 overlapping nonamers. To screen this protein for potential epitopes, 33 epitopes would need to be experimentally checked for each MHC molecule of interest if rescaled predictions were used, as opposed to 20 for the non-rescaled predictions (providing 85% epitope coverage was sufficient).

Added to the significant results from the ROC curve analysis, the supplementary analysis demonstrated the positive effect of removing rescaling in terms of the correlation with experimental data (supplementary figure S3) and also in terms of per-protein and sensitivity analysis (supplementary figure S2 and tables S1 and S2). Taken together, these results strongly demonstrate the improvement in accuracy of removing the condition of rescaling when comparing predictions between alleles.

There has been little research on the variation in ‘stickiness’ among MHC molecules, i.e. whether some MHC class I molecules are capable of binding to a greater number of epitopes than others. The binding motifs for MHC-peptide binding vary across the range of alleles, but the assumption made for rescaling is that each molecule would bind to the same number of peptides out of a large random selection. Estimates based upon mass spectrometry suggest that over 2,000 peptides are associated with HLA-A2.1 and −B7 and it is speculated that the actual total could be over 10,000 per MHC molecule [36]. However, it is not known how this number varies between molecules. It has been postulated that the twin constraints of effective pathogen recognition but tolerance of self would result in a very narrow range of promiscuity for viable MHC class I molecules [29]. Contrary to this, recent research has shown that this range may be wider than initially envisaged [37] and our results suggest that there is considerable inter-allelic variation in promiscuity.

This data may also be informative regarding optimization of peptide cargo in the endoplasmic reticulum (ER). We would argue that peptide optimization is the biological interpretation of rescaling: alleles have similar numbers of epitopes because peptides with a lower binding affinity are replaced in the ER. We know that optimisation cannot be complete because otherwise every allele would just present one epitope: the one with highest affinity. However, it seems likely that there is a degree of optimization [30],[31]. The observation that rescaling gives worse predictions may put a bound on how much optimisation is occurring. Allied to this, it has been observed that the release of an MHC class I molecule from the peptide-loading complex with a *suboptimal peptide* takes precedence over the prolonged detention of the MHC class I molecule in the complex until an optimal peptide comes along [30]. Hence, peptide optimization acts to reduce inter-allelic variation and promiscuity results from inter-allelic variation in allele-peptide affinity. However, this peptide optimization is limited by time and is not complete and hence, we note this variation in promiscuity across different alleles.

In summary, we suggest that much of the observed variation between allelic predictors reflects genuine biological information which should not be discarded as experimental noise and that rescaling is based on an unjustified assumption: that all alleles bind the same number of peptides. Removing this assumption, we have demonstrated a significantly improved predictive performance.

These conclusions are important both for studies that use prediction methods to understand the CTL response and for T cell epitope discovery programs where avoiding rescaling could save a large amount of experimental effort, ultimately leading to improved vaccine implementation.

## Supporting Information

Dataset S1 The HIV HXB2 proteome.
(0.06 MB DOC)

Dataset S2 The SYF1 dataset.
(0.14 MB DOC)

Dataset S3 The Lanl 661 dataset.
(0.54 MB DOC)

Dataset S4 The Lanl 179 dataset.
(0.16 MB DOC)

Table S1 The specificity of non-rescaled and rescaled results at specified sensitivity values.
(0.03 MB DOC)

Table S2 The fraction of the total number of epitopes in the 2 epitope datasets among the top 5% of predicted binding affinities.
(0.03 MB DOC)

Figure S1 The result of the ROC curve analysis, using the Lanl 661 dataset and excluding any alleles (7 in total) that had an AUC<0.9 from figure 2 (bootstrap: p<0.001).
(0.03 MB DOC)

Figure S2 A comparison of ranks between rescaled and non-rescaled predicted binding affinities.
(0.03 MB DOC)

Figure S3 The relationship between rescaled/non-rescaled predicted binding affinities and experimental binding affinities.
(0.19 MB DOC)

Figure S4 A comparison of rescale values.
(0.04 MB DOC)

## References

[1] Yewdell JW, Bennink JR (1999). Immunodominance in major histocompatibility complex class I-restricted T lymphocyte responses.. Annu Rev Immunol.

[2] Assarsson E, Sidney J, Oseroff C, Pasquetto V, Bui HH (2007). A quantitative analysis of the variables affecting the repertoire of T cell specificities recognized after vaccinia virus infection.. J Immunol.

[3] Larsen MV, Lundegaard C, Lamberth K, Buus S, Brunak S (2005). An integrative approach to CTL epitope prediction: a combined algorithm integrating MHC class I binding, TAP transport efficiency, and proteasomal cleavage predictions.. Eur J Immunol.

[4] Carrington M, Nelson GW, Martin MP, Kissner T, Vlahov D (1999). HLA and HIV-1: heterozygote advantage and B*35-Cw*04 disadvantage.. Science.

[5] Bangham CRM, Osame M (2005). Cellular immune response to HTLV-1.. Oncogene.

[6] Bui HH, Sidney J, Dinh K, Southwood S, Newman MJ (2006). Predicting population coverage of T-cell epitope-based diagnostics and vaccines.. BMC Bioinformatics.

[7] Sette A, Fleri W, Peters B, Sathiamurthy M, Bui HH (2005). A roadmap for the immunomics of category A-C pathogens.. Immunity.

[8] Sette A, Peters B (2007). Immune epitope mapping in the post-genomic era: lessons for vaccine development.. Curr Opin Immunol.

[9] Snyder JT, Belyakov IM, Dzutsev A, Lemonnier F, Berzofsky JA (2004). Protection against lethal vaccinia virus challenge in HLA-A2 transgenic mice by immunization with a single CD8+ T-cell peptide epitope of vaccinia and variola viruses.. J Virol.

[10] Drexler I, Staib C, Kastenmuller W, Stevanović S, Schmidt B (2003). Identification of vaccinia virus epitope-specific HLA-A*0201-restricted T cells and comparative analysis of smallpox vaccines.. Proc Natl Acad Sci U S A.

[11] Oseroff C, Kos F, Bui HH, Peters B, Pasquetto V (2005). HLA class I-restricted responses to vaccinia recognize a broad array of proteins mainly involved in virulence and viral gene regulation.. Proc Natl Acad Sci U S A.

[12] Pasquetto V, Bui HH, Giannino R, Banh C, Mirza F (2005). HLA-A*0201, HLA-A*1101, and HLA-B*0702 transgenic mice recognize numerous poxvirus determinants from a wide variety of viral gene products.. J Immunol.

[13] Wang M, Johansen B, Nissen MH, Thorn M, Kløverpris H (2007). Identification of an HLA-A*0201 restricted Bcl2-derived epitope expressed on tumors.. Cancer Lett.

[14] Thorn M, Wang M, Kløverpris H, Schmidt EGW, Fomsgaard A (2007). Identification of a new hTERT-derived HLA-A*0201 restricted, naturally processed CTL epitope.. Cancer Immunol Immunother.

[15] Wang M, Lamberth K, Harndahl M, Røder G, Stryhn A (2007). CTL epitopes for influenza A including the H5N1 bird flu; genome-, pathogen-, and HLA-wide screening.. Vaccine.

[16] Moutaftsi M, Peters B, Pasquetto V, Tscharke DC, Sidney J (2006). A consensus epitope prediction approach identifies the breadth of murine T(CD8+)-cell responses to vaccinia virus.. Nat Biotechnol.

[17] Brumme ZL, Brumme CJ, Heckerman D, Korber BT, Daniels M (2007). Evidence of Differential HLA Class I-Mediated Viral Evolution in Functional and Accessory/Regulatory Genes of HIV-1.. PLoS Pathog.

[18] Borghans JAM, Mølgaard A, de Boer RJ, Keşmir C (2007). HLA Alleles Associated with Slow Progression to AIDS Truly Prefer to Present HIV-1 p24.. PLoS ONE.

[19] Rolland M, Heckerman D, Deng W, Rousseau CM, Coovadia H (2008). Broad and Gag-Biased HIV-1 Epitope Repertoires Are Associated with Lower Viral Loads.. PLoS ONE.

[20] Rammensee H, Bachmann J, Emmerich NP, Bachor OA, Stevanović S (1999). SYFPEITHI: database for MHC ligands and peptide motifs.. Immunogenetics.

[21] Nielsen M, Lundegaard C, Worning P, Hvid CS, Lamberth K (2004). Improved prediction of MHC class I and class II epitopes using a novel Gibbs sampling approach.. Bioinformatics.

[22] Bui HH, Sidney J, Peters B, Sathiamurthy M, Sinichi A (2005). Automated generation and evaluation of specific MHC binding predictive tools: ARB matrix applications.. Immunogenetics.

[23] Peters B, Sette A (2005). Generating quantitative models describing the sequence specificity of biological processes with the stabilized matrix method.. BMC Bioinformatics.

[24] Heckerman D, Kadie C, Listgarten J (2007). Leveraging information across HLA alleles/supertypes improves epitope prediction.. J Comput Biol.

[25] Buus S, Lauemøller SL, Worning P, Kesmir C, Frimurer T (2003). Sensitive quantitative predictions of peptide-MHC binding by a ‘Query by Committee’ artificial neural network approach.. Tissue Antigens.

[26] Nielsen M, Lundegaard C, Worning P, Lauemøller SL, Lamberth K (2003). Reliable prediction of T-cell epitopes using neural networks with novel sequence representations.. Protein Sci.

[27] Peters B, Bui HH, Sidney J, Weng Z, Loffredo JT (2005). A computational resource for the prediction of peptide binding to Indian rhesus macaque MHC class I molecules.. Vaccine.

[28] Sturniolo T, Bono E, Ding J, Raddrizzani L, Tuereci O (1999). Generation of tissue-specific and promiscuous HLA ligand databases using DNA microarrays and virtual HLA class II matrices.. Nat Biotechnol.

[29] George AJT, Stark J, Chan C (2005). Understanding specificity and sensitivity of T-cell recognition.. Trends Immunol.

[30] Elliott T (2006). The ‘chop-and-change’ of MHC class I assembly.. Nat Immunol.

[31] Williams AP, Peh CA, Purcell AW, McCluskey J, Elliott T (2002). Optimization of the MHC class I peptide cargo is dependent on tapasin.. Immunity.

[32] Sette A, Sidney J (1999). Nine major HLA class I supertypes account for the vast preponderance of HLA-A and -B polymorphism.. Immunogenetics.

[33] Korber BTM, Brander C, Haynes BF, Koup R, Moore JP (2005). HIV Molecular Immunology 2005.

[34] Peters B, Bui HH, Frankild S, Nielson M, Lundegaard C (2006). A community resource benchmarking predictions of peptide binding to MHC-I molecules.. PLoS Comput Biol.

[35] Larsen M, Lundegaard C, Lamberth K, Buus S, Lund O (2007). Large-Scale validation of methods for cytotoxic T-lymphocyte epitope prediction.. BMC Bioinformatics.

[36] Engelhard VH (1994). Structure of peptides associated with class I and class II MHC molecules.. Annu Rev Immunol.

[37] Frahm N, Yusim K, Suscovich TJ, Adams S, Sidney J (2007). Extensive HLA class I allele promiscuity among viral CTL epitopes.. Eur J Immunol.

