# Supplementary material for: T-Cell Epitope Prediction: Rescaling Can Mask Biological Variation between MHC Molecules
Source: PLoS Comput Biol. 2009 Mar 20;5(3):e1000327. doi: 10.1371/journal.pcbi.1000327 (PMC2650421; doi:10.1371/journal.pcbi.1000327)
Supplement: Dataset S1 — The HIV HXB2 proteome. (0.06 MB DOC) [file pcbi.1000327.s001.doc]

**SYF^1^**

Dataset S2: The SYF^1^ dataset

| Epitope | Allele | Protein | Epitope Start |
| --- | --- | --- | --- |
| AYSSWMYSY | A1 | EBN3_EBV | 44 |
| AIVDKVPSV | A2 | COPG_HUMAN | 147 |
| ALADGVQKV | A2 | APL1_HUMAN | 160 |
| ALANGIEEV | A2 | APL3_HUMAN | 172 |
| ALASHLIEA | A2 | EHD2_HUMAN | 507 |
| ALFGALFLA | A2 | PLTP_HUMAN | 2 |
| ALLNIKVKL | A2 | K1CR_HUMAN | 364 |
| ALLVLYSFA | A2 | LMP1_EBV | 51 |
| ALPHAILRL | A2 | ACTB_HUMAN | 170 |
| FLALIICNA | A2 | MSHR_HUMAN | 283 |
| FLDGNELTL | A2 | CLI1_HUMAN | 167 |
| FLDGNEMTL | A2 | CLI4_HUMAN | 178 |
| FLDPRPLTV | A2 | CP1B_HUMAN | 190 |
| FLLDKKIGV | A2 | TCPB_HUMAN | 218 |
| GLIEKNIEL | A2 | DNM1_HUMAN | 425 |
| GLLGTLVQL | A2 | CTNB_HUMAN | 400 |
| GLYPGLIWL | A2 | IRF6_HUMAN | 21 |
| KASEKIFYV | A2 | SSX2_HUMAN | 41 |
| LLFDRPMHV | A2 | ROM_HUMAN | 268 |
| LLMGTLGIV | A2 | VE7_HPV16 | 82 |
| LTAGFLIFL | A2 | LMP2_EBV | 453 |
| NLLPKLHIV | A2 | CLI1_HUMAN | 179 |
| NLTISDVSV | A2 | MUC1_HUMAN | 1133 |
| QLIDKVWQL | A2 | SC14_HUMAN | 593 |
| RLVDDFLLV | A2 | TERT_HUMAN | 865 |
| SLFPGKLEV | A2 | FLIH_HUMAN | 1010 |
| SLIGHLQTL | A2 | DUS5_HUMAN | 337 |
| SLSEKTVLL | A2 | CD59_HUMAN | 106 |
| SLWGQPAEA | A2 | CA54_HUMAN | 18 |
| STAPPVHNV | A2 | MUC1_HUMAN | 950 |
| SVASTITGV | A2 | ADFP_HUMAN | 129 |
| SVFAGVVGV | A2 | CYG3_HUMAN | 581 |
| TIHDIILEC | A2 | VE6_HPV16 | 29 |
| TILLGIFFL | A2 | MSHR_HUMAN | 244 |
| VLEETSVML | A2 | VIE1_HCMVA | 316 |
| VMAPRTLVL | A2 | 1A23_HUMAN | 3 |
| WLNEVEFKL | A2 | DMD_HUMAN | 1281 |
| YLDNGVVFV | A2 | DDB1_HUMAN | 316 |
| YLVTRHADV | A2 | POLG_HCVH | 1131 |
| YVDPVITSI | A2 | MET_HUMAN | 654 |
| DADKYAVTV | B7 | OM1E_CHLTR | 367 |
| DAEMTTRMV | B7 | PSBA_HUMAN | 90 |
| DAENAMRYI | B7 | CB20_HUMAN | 93 |
| DALLIIPKV | B7 | TCPW_HUMAN | 441 |
| DALLKFSHI | B7 | BI1_HUMAN | 11 |
| DALLQMITI | B7 | EF2_HUMAN | 347 |
| DALRSILTI | B7 | SYM_HUMAN | 703 |
| DAYVLPKLY | B7 | RS26_HUMAN | 60 |
| DGYEQAARV | B7 | TCPE_HUMAN | 135 |
| DPYEVSYRI | B7 | BTG1_HUMAN | 107 |
| DPYKVYRIV | B7 | IRF4_HUMAN | 120 |
| FAYVQIKTI | B7 | CP51_HUMAN | 454 |
| HPDIVIYQY | B7 | POL_HV1U4 | 329 |
| IPQQHTQVL | B7 | CEA5_HUMAN | 632 |
| KPAFFAEKL | B7 | ANX1_HUMAN | 273 |
| KPSLPFTSL | B7 | CYRG_HUMAN | 3 |
| LPRSTVINI | B7 | IFM1_HUMAN | 19 |
| MPMNVADLI | B7 | IF42_HUMAN | 399 |
| MPWFKGWKV | B7 | EF11_HUMAN | 208 |
| NAACMALNI | B7 | OM1E_CHLTR | 121 |
| NAYEYFTKI | B7 | BAK2_HUMAN | 106 |
| NAYVNINRI | B7 | HAPP_HUMAN | 363 |
| NPVPVGNIY | B7 | GAG_HV2G1 | 257 |
| NSSKVSQNY | B7 | GAG_HV1J3 | 123 |
| PPIPVGDIY | B7 | GAG_HV1MA | 259 |
| PPSGKGGNY | B7 | GAG_HV2CA | 127 |
| SPKLPVSSL | B7 | DRI1_HUMAN | 372 |
| SPYQNIKIL | B7 | SPSY_HUMAN | 145 |
| TSEHSHFSL | B7 | DCE2_HUMAN | 277 |
| TVLDVGDAY | B7 | POL_HV1BR | 274 |
| VFPTKDVAL | B7 | PP65_HCMVA | 187 |
| VPSEPGGVL | B7 | PTN6_HUMAN | 422 |
| WASRELERF | B7 | GAG_HV1BR | 35 |
| YAFNMKATV | B7 | HS7C_HUMAN | 545 |
| ELRRKMMYM | B8 | VIE1_HCMVA | 199 |
| ELRSRYWAI | B8 | VNUC_IAPUE | 380 |
| GEIYKRWII | B8 | GAG_HV1BR | 258 |
| VMLRWGVLA | B8 | NCAP_HRSVA | 256 |
| WVKEKVVAL | B8 | NK4_HUMAN | 175 |
| ARFGLIQSM | B27 | Y174_HUMAN | 81 |
| GRFSGLLGR | B27 | IL16_HUMAN | 180 |
| GRNVVLDKS | B27 | CH60_YEREN | 35 |
| IRAAPPPLF | B27 | PRTP_HUMAN | 2 |
| IRAASAITA | B27 | CH60_YEREN | 420 |
| IRGAIILAK | B27 | RS2_HUMAN | 151 |
| IRLRPGGKK | B27 | GAG_HV1BR | 18 |
| IRNDEELNK | B27 | H2AC_HUMAN | 87 |
| IRRGVMLAV | B27 | CH60_HUMAN | 140 |
| KRGIDKAVI | B27 | CH60_YEREN | 117 |
| KRIQEIIEQ | B27 | CH60_HUMAN | 369 |
| KRTLKIPAM | B27 | CH60_HUMAN | 469 |
| MRMATPLLM | B27 | HG2A_HUMAN | 107 |
| NRIVYLYTK | B27 | RL34_HUMAN | 27 |
| QRNLYIAGF | B27 | CDM_HUMAN | 100 |
| QRNVNIFKF | B27 | LDHA_HUMAN | 110 |
| QRVNVQPEL | B27 | PGTB_HUMAN | 321 |
| TRYQGVNLY | B27 | PAB1_HUMAN | 289 |
| AENLWVTVY | B44 | ENV_HV1S3 | 30 |
| AETPDIKLF | B44 | RS5_HUMAN | 12 |
| NEGLGWAGW | B44 | POLG_HCVJ6 | 88 |
| IALYLQQNW | B58 | LMP1_EBV | 156 |
| ITTKAISRW | B58 | TCPG_HUMAN | 159 |
| KTKEVIQEW | B58 | TALI_HUMAN | 343 |
| VSFIEFVGW | B58 | EBN4_EBV | 279 |
| AFHHVAREL | B62 | NEF_HV1BR | 190 |
| GFYPGSIEV | B62 | HB2I_HUMAN | 150 |
| IKADHVSTY | B62 | HA2Q_HUMAN | 32 |
| WQYFFPVIF | B62 | MAG3_HUMAN | 143 |
| DTAAQITQR | A3 | 1B35_HUMAN | 161 |
| TIIDILTKR | A3 | ANX1_HUMAN | 63 |
| TIVNILTNR | A3 | ANX2_HUMAN | 54 |
| TIIDIITHR | A3 | ANX6_HUMAN | 384 |
| ATIGTAMYK | A3 | BRL1_EBV | 134 |
| GSPATWTTR | A3 | CA34_HUMAN | 1436 |
| YVNVNMGLK | A3 | CORA_HPBV4 | 88 |
| RFKMFPEVK | A3 | DCE2_HUMAN | 255 |
| TLYCVHQRI | A3 | GAG_HV1BR | 83 |
| IVGLNKIVR | A3 | GAG_HV1EL | 266 |
| DVFVVGTER | A3 | GTFI_HUMAN | 53 |
| SIMKWNRER | A3 | NB6M_HUMAN | 48 |
| ALNFPGSQK | A3 | PM17_HUMAN | 87 |
| RLGVRATRK | A3 | POLG_HCV1 | 43 |
| GPISGHVLK | A3 | PP65_HCMVA | 16 |
| YTPTISRER | A3 | PSB2_HUMAN | 147 |
| QAIKGMHIR | A3 | RL17_HUMAN | 34 |
| SLADIMAKR | A3 | RL24_HUMAN | 86 |
| DTIEIITDR | A3 | ROA2_HUMAN | 139 |
| ETIGEILKK | A3 | ROK_HUMAN | 95 |
| FCVGFTKKR | A3 | RS3A_HUMAN | 137 |
| EVVVSGKLR | A3 | RS3_HUMAN | 135 |
| ETFSGVYKK | A3 | RS7_HUMAN | 171 |
| STIEYVIQR | A3 | S23B_HUMAN | 115 |
| TPAGGGFPR | A3 | TISB_HUMAN | 43 |
| AIYKQSQHM | A24 | P53_HUMAN | 161 |
| AYSQQTRGL | A24 | POLG_HCVBK | 1031 |
| EYLQLVFGI | A24 | MAG2_HUMAN | 156 |
| EYLVSFGVW | A24 | CORA_HPBVJ | 117 |
| HYTNASDGL | A24 | LCK_HUMAN | 207 |
| KYTSFPWLL | A24 | DPOL_HPBVJ | 756 |
| QFQSIYAKF | A24 | RECO_HUMAN | 47 |
| QYDPVAALF | A24 | PP65_HCMVA | 341 |
| RWPSCQKKF | A24 | WT1_HUMAN | 417 |
| TFDYLRSVL | A24 | LCK_HUMAN | 485 |
| TYGEIFEKF | A24 | N4BM_HUMAN | 107 |
| VYAETKHFL | A24 | TERT_HUMAN | 324 |
| VYALPLKML | A24 | PP65_HCMVA | 113 |
| VYGFVRACL | A24 | TERT_HUMAN | 461 |
| YYMIGEQKF | A24 | NNMT_HUMAN | 203 |
