# Supplementary material for: T-Cell Epitope Prediction: Rescaling Can Mask Biological Variation between MHC Molecules
Source: PLoS Comput Biol. 2009 Mar 20;5(3):e1000327. doi: 10.1371/journal.pcbi.1000327 (PMC2650421; doi:10.1371/journal.pcbi.1000327)
Supplement: Dataset S2 — The SYF1 dataset. (0.14 MB DOC) [file pcbi.1000327.s002.doc]

**HIV HXB2**

Dataset S1: The HIV HXB2 proteome

| >gp160 |
| --- |
| MRVKEKYQHLWRWGWRWGTMLLGMLMICSATEKLWVTVYYGVPVWKEATTTLFCASDAKAYDTEVHNVWATHACVPTDPNPQEVVLVNVTENFNMWKNDM |
| VEQMHEDIISLWDQSLKPCVKLTPLCVSLKCTDLKNDTNTNSSSGRMIMEKGEIKNCSFNISTSIRGKVQKEYAFFYKLDIIPIDNDTTSYKLTSCNTSV |
| ITQACPKVSFEPIPIHYCAPAGFAILKCNNKTFNGTGPCTNVSTVQCTHGIRPVVSTQLLLNGSLAEEEVVIRSVNFTDNAKTIIVQLNTSVEINCTRPN |
| NNTRKRIRIQRGPGRAFVTIGKIGNMRQAHCNISRAKWNNTLKQIASKLREQFGNNKTIIFKQSSGGDPEIVTHSFNCGGEFFYCNSTQLFNSTWFNSTW |
| STEGSNNTEGSDTITLPCRIKQIINMWQKVGKAMYAPPISGQIRCSSNITGLLLTRDGGNSNNESEIFRPGGGDMRDNWRSELYKYKVVKIEPLGVAPTK |
| AKRRVVQREKRAVGIGALFLGFLGAAGSTMGAASMTLTVQARQLLSGIVQQQNNLLRAIEAQQHLLQLTVWGIKQLQARILAVERYLKDQQLLGIWGCSG |
| KLICTTAVPWNASWSNKSLEQIWNHTTWMEWDREINNYTSLIHSLIEESQNQQEKNEQELLELDKWASLWNWFNITNWLWYIKLFIMIVGGLVGLRIVFA |
| VLSIVNRVRQGYSPLSFQTHLPTPRGPDRPEGIEEEGGERDRDRSIRLVNGSLALIWDDLRSLCLFSYHRLRDLLLIVTRIVELLGRRGWEALKYWWNLL |
| QYWSQELKNSAVSLLNATAIAVAEGTDRVIEVVQGACRAIRHIPRRIRQGLERILL |
| >Integrase |
| FLDGIDKAQDEHEKYHSNWRAMASDFNLPPVVAKEIVASCDKCQLKGEAMHGQVDCSPGIWQLDCTHLEGKVILVAVHVASGYIEAEVIPAETGQETAYF |
| LLKLAGRWPVKTIHTDNGSNFTGATVRAACWWAGIKQEFGIPYNPQSQGVVESMNKELKKIIGQVRDQAEHLKTAVQMAVFIHNFKRKGGIGGYSAGERI |
| VDIIATDIQTKELQKQITKIQNFRVYYRDSRNPLWKGPAKLLWKGEGAVVIQDNSDIKVVPRRKAKIIRDYGKQMAGDDCVASRQDED |
| >Nef |
| MGGKWSKSSVIGWPTVRERMRRAEPAADRVGAASRDLEKHGAITSSNTAATNAACAWLEAQEEEEVGFPVTPQVPLRPMTYKAAVDLSHFLKEKGGLEGL |
| IHSQRRQDILDLWIYHTQGYFPDWQNYTPGPGVRYPLTFGWCYKLVPVEPDKIEEANKGENTSLLHPVSLHGMDDPEREVLEWRFDSRLAFHHVARELHP |
| EYFKNC |
| >p17 |
| MGARASVLSGGELDRWEKIRLRPGGKKKYKLKHIVWASRELERFAVNPGLLETSEGCRQILGQLQPSLQTGSEELRSLYNTVATLYCVHQRIEIKDTKEA |
| LDKIEEEQNKSKKKAQQAAADTGHSNQVSQNY |
| >p24-p2p7p1p6 |
| PIVQNIQGQMVHQAISPRTLNAWVKVVEEKAFSPEVIPMFSALSEGATPQDLNTMLNTVGGHQAAMQMLKETINEEAAEWDRVHPVHAGPIAPGQMREPR |
| GSDIAGTTSTLQEQIGWMTNNPPIPVGEIYKRWIILGLNKIVRMYSPTSILDIRQGPKEPFRDYVDRFYKTLRAEQASQEVKNWMTETLLVQNANPDCKT |
| ILKALGPAATLEEMMTACQGVGGPGHKARVLAEAMSQVTNSATIMMQRGNFRNQRKIVKCFNCGKEGHTARNCRAPRKKGCWKCGKEGHQMKDCTERQAN |
| FLGKIWPSYKGRPGNFLQSRPEPTAPPEESFRSGVETTTPPQKQEPIDKELYPLTSLRSLFGNDPSSQ |
| >Protease |
| PQVTLWQRPLVTIKIGGQLKEALLDTGADDTVLEEMSLPGRWKPKMIGGIGGFIKVRQYDQILIEICGHKAIGTVLVGPTPVNIIGRNLLTQIGCTLNF |
| >Rev |
| MAGRSGDSDEELIRTVRLIKLLYQSNPPPNPEGTRQARRNRRRRWRERQRQIHSISERILGTYLGRSAEPVPLQLPPLERLTLDCNEDCGTSGTQGVGSP |
| QILVESPTVLESGTKE |
| >RT |
| PISPIETVPVKLKPGMDGPKVKQWPLTEEKIKALVEICTEMEKEGKISKIGPENPYNTPVFAIKKKDSTKWRKLVDFRELNKRTQDFWEVQLGIPHPAGL |
| KKKKSVTVLDVGDAYFSVPLDEDFRKYTAFTIPSINNETPGIRYQYNVLPQGWKGSPAIFQSSMTKILEPFRKQNPDIVIYQYMDDLYVGSDLEIGQHRT |
| KIEELRQHLLRWGLTTPDKKHQKEPPFLWMGYELHPDKWTVQPIVLPEKDSWTVNDIQKLVGKLNWASQIYPGIKVRQLCKLLRGTKALTEVIPLTEEAE |
| LELAENREILKEPVHGVYYDPSKDLIAEIQKQGQGQWTYQIYQEPFKNLKTGKYARMRGAHTNDVKQLTEAVQKITTESIVIWGKTPKFKLPIQKETWET |
| WWTEYWQATWIPEWEFVNTPPLVKLWYQLEKEPIVGAETFYVDGAANRETKLGKAGYVTNRGRQKVVTLTDTTNQKTELQAIYLALQDSGLEVNIVTDSQ |
| YALGIIQAQPDQSESELVNQIIEQLIKKEKVYLAWVPAHKGIGGNEQVDKLVSAGIRKVL |
| >Tat |
| MEPVDPRLEPWKHPGSQPKTACTNCYCKKCCFHCQVCFITKALGISYGRKKRRQRRRAHQNSQTHQASLSKQPTSQPRGDPTGPKEWKKKVERETETDPF |
| D |
| >Vif |
| MENRWQVMIVWQVDRMRIRTWKSLVKHHMYVSGKARGWFYRHHYESPHPRISSEVHIPLGDARLVITTYWGLHTGERDWHLGQGVSIEWRKKRYSTQVDP |
| ELADQLIHLYYFDCFSDSAIRKALLGHIVSPRCEYQAGHNKVGSLQYLALAALITPKKIKPPLPSVTKLTEDRWNKPQKTKGHRGSHTMNGH |
| >Vpr |
| MEQAPEDQGPQREPHNEWTLELLEELKNEAVRHFPRIWLHGLGQHIYETYGDTWAGVEAIIRILQQLLFIHFRIGCRHSRIGVTRQRRARNGASRS |
| >Vpu |
| TQPIPIVAIVALVVAIIIAIVVWSIVIIEYRKILRQRKIDRLIDRLIERAEDSGNESEGEISALVEMGVEMGHHAPWDVDDL |
