# Supplementary material for: T-Cell Epitope Prediction: Rescaling Can Mask Biological Variation between MHC Molecules
Source: PLoS Comput Biol. 2009 Mar 20;5(3):e1000327. doi: 10.1371/journal.pcbi.1000327 (PMC2650421; doi:10.1371/journal.pcbi.1000327)
Supplement: Dataset S3 — The Lanl 661 dataset. (0.54 MB DOC) [file pcbi.1000327.s003.doc]

**Lanl^661^**

Dataset S3: The Lanl^661^ dataset

| Epitope | Allele | Protein | HXB2 Start |
| --- | --- | --- | --- |
| RRGWEALKY | A0101 | gp160 | 787 |
| WIYHTQGYF | A0101 | Nef | 113 |
| YFPDWQNYT | A0101 | Nef | 120 |
| GSEELRSLY | A0101 | p17 | 71 |
| QRPLVTIKI | A0101 | Protease | 7 |
| ISERILGTY | A0101 | Rev | 55 |
| LWVTVYYGV | A0201 | gp160 | 34 |
| VTVYYGVPV | A0201 | gp160 | 36 |
| NVWATHACV | A0201 | gp160 | 67 |
| QMHEDIISL | A0201 | gp160 | 103 |
| KLTPLCVSL | A0201 | gp160 | 121 |
| KLTSCNTSV | A0201 | gp160 | 192 |
| QRGPGRAFV | A0201 | gp160 | 310 |
| TLKQIASKL | A0201 | gp160 | 341 |
| TMGAASMTL | A0201 | gp160 | 529 |
| AVLSIVNRV | A0201 | gp160 | 700 |
| RLVNGSLAL | A0201 | gp160 | 747 |
| RLRDLLLIV | A0201 | gp160 | 770 |
| LLNATAIAV | A0201 | gp160 | 814 |
| RVIEVVQGA | A0201 | gp160 | 828 |
| RIRQGLERI | A0201 | gp160 | 846 |
| QVRDQAEHL | A0201 | Integrase | 164 |
| LLWKGEGAV | A0201 | Integrase | 241 |
| ATNAACAWL | A0201 | Nef | 50 |
| AAVDLSHFL | A0201 | Nef | 83 |
| YPLTFGWCY | A0201 | Nef | 135 |
| LTFGWCYKL | A0201 | Nef | 137 |
| LEWRFDSRL | A0201 | Nef | 181 |
| AFHHVAREL | A0201 | Nef | 190 |
| SLYNTVATL | A0201 | p17 | 77 |
| TLNAWVKVV | A0201 | p24-p2p7p1 | 19 |
| AMQMLKETI | A0201 | p24-p2p7p1 | 65 |
| AEWDRVHPV | A0201 | p24-p2p7p1 | 78 |
| TLQEQIGWM | A0201 | p24-p2p7p1 | 110 |
| MTNNPPIPV | A0201 | p24-p2p7p1 | 118 |
| RMYSPTSIL | A0201 | p24-p2p7p1 | 143 |
| YVDRFYKTL | A0201 | p24-p2p7p1 | 164 |
| VLAEAMSQV | A0201 | p24-p2p7p1 | 230 |
| LVGPTPVNI | A0201 | Protease | 76 |
| ALVEICTEM | A0201 | RT | 33 |
| YTAFTIPSI | A0201 | RT | 127 |
| VIYQYMDDL | A0201 | RT | 179 |
| YQYMDDLYV | A0201 | RT | 181 |
| KIEELRQHL | A0201 | RT | 201 |
| ILKEPVHGV | A0201 | RT | 309 |
| PLVKLWYQL | A0201 | RT | 421 |
| KLGKAGYVT | A0201 | RT | 451 |
| ALQDSGLEV | A0201 | RT | 485 |
| AIIRILQQL | A0201 | Vpr | 59 |
| RILQQLLFI | A0201 | Vpr | 62 |
| VVAIIIAIV | A0201 | Vpu | 13 |
| LWVTVYYGV | A0202 | gp160 | 34 |
| VTVYYGVPV | A0202 | gp160 | 36 |
| NVWATHACV | A0202 | gp160 | 67 |
| QMHEDIISL | A0202 | gp160 | 103 |
| KLTPLCVSL | A0202 | gp160 | 121 |
| KLTSCNTSV | A0202 | gp160 | 192 |
| QRGPGRAFV | A0202 | gp160 | 310 |
| TLKQIASKL | A0202 | gp160 | 341 |
| TMGAASMTL | A0202 | gp160 | 529 |
| AVLSIVNRV | A0202 | gp160 | 700 |
| RLVNGSLAL | A0202 | gp160 | 747 |
| RLRDLLLIV | A0202 | gp160 | 770 |
| LLNATAIAV | A0202 | gp160 | 814 |
| RVIEVVQGA | A0202 | gp160 | 828 |
| RIRQGLERI | A0202 | gp160 | 846 |
| QVRDQAEHL | A0202 | Integrase | 164 |
| LLWKGEGAV | A0202 | Integrase | 241 |
| ATNAACAWL | A0202 | Nef | 50 |
| AAVDLSHFL | A0202 | Nef | 83 |
| YPLTFGWCY | A0202 | Nef | 135 |
| LTFGWCYKL | A0202 | Nef | 137 |
| LEWRFDSRL | A0202 | Nef | 181 |
| AFHHVAREL | A0202 | Nef | 190 |
| SLYNTVATL | A0202 | p17 | 77 |
| TLNAWVKVV | A0202 | p24-p2p7p1 | 19 |
| AMQMLKETI | A0202 | p24-p2p7p1 | 65 |
| AEWDRVHPV | A0202 | p24-p2p7p1 | 78 |
| TLQEQIGWM | A0202 | p24-p2p7p1 | 110 |
| MTNNPPIPV | A0202 | p24-p2p7p1 | 118 |
| RMYSPTSIL | A0202 | p24-p2p7p1 | 143 |
| YVDRFYKTL | A0202 | p24-p2p7p1 | 164 |
| VLAEAMSQV | A0202 | p24-p2p7p1 | 230 |
| LVGPTPVNI | A0202 | Protease | 76 |
| ALVEICTEM | A0202 | RT | 33 |
| YTAFTIPSI | A0202 | RT | 127 |
| VIYQYMDDL | A0202 | RT | 179 |
| YQYMDDLYV | A0202 | RT | 181 |
| KIEELRQHL | A0202 | RT | 201 |
| ILKEPVHGV | A0202 | RT | 309 |
| PLVKLWYQL | A0202 | RT | 421 |
| KLGKAGYVT | A0202 | RT | 451 |
| ALQDSGLEV | A0202 | RT | 485 |
| AIIRILQQL | A0202 | Vpr | 59 |
| RILQQLLFI | A0202 | Vpr | 62 |
| VVAIIIAIV | A0202 | Vpu | 13 |
| LWVTVYYGV | A0203 | gp160 | 34 |
| VTVYYGVPV | A0203 | gp160 | 36 |
| NVWATHACV | A0203 | gp160 | 67 |
| QMHEDIISL | A0203 | gp160 | 103 |
| KLTPLCVSL | A0203 | gp160 | 121 |
| KLTSCNTSV | A0203 | gp160 | 192 |
| QRGPGRAFV | A0203 | gp160 | 310 |
| TLKQIASKL | A0203 | gp160 | 341 |
| TMGAASMTL | A0203 | gp160 | 529 |
| AVLSIVNRV | A0203 | gp160 | 700 |
| RLVNGSLAL | A0203 | gp160 | 747 |
| RLRDLLLIV | A0203 | gp160 | 770 |
| LLNATAIAV | A0203 | gp160 | 814 |
| RVIEVVQGA | A0203 | gp160 | 828 |
| RIRQGLERI | A0203 | gp160 | 846 |
| QVRDQAEHL | A0203 | Integrase | 164 |
| LLWKGEGAV | A0203 | Integrase | 241 |
| ATNAACAWL | A0203 | Nef | 50 |
| AAVDLSHFL | A0203 | Nef | 83 |
| YPLTFGWCY | A0203 | Nef | 135 |
| LTFGWCYKL | A0203 | Nef | 137 |
| LEWRFDSRL | A0203 | Nef | 181 |
| AFHHVAREL | A0203 | Nef | 190 |
| SLYNTVATL | A0203 | p17 | 77 |
| TLNAWVKVV | A0203 | p24-p2p7p1 | 19 |
| AMQMLKETI | A0203 | p24-p2p7p1 | 65 |
| AEWDRVHPV | A0203 | p24-p2p7p1 | 78 |
| TLQEQIGWM | A0203 | p24-p2p7p1 | 110 |
| MTNNPPIPV | A0203 | p24-p2p7p1 | 118 |
| RMYSPTSIL | A0203 | p24-p2p7p1 | 143 |
| YVDRFYKTL | A0203 | p24-p2p7p1 | 164 |
| VLAEAMSQV | A0203 | p24-p2p7p1 | 230 |
| LVGPTPVNI | A0203 | Protease | 76 |
| ALVEICTEM | A0203 | RT | 33 |
| YTAFTIPSI | A0203 | RT | 127 |
| VIYQYMDDL | A0203 | RT | 179 |
| YQYMDDLYV | A0203 | RT | 181 |
| KIEELRQHL | A0203 | RT | 201 |
| ILKEPVHGV | A0203 | RT | 309 |
| PLVKLWYQL | A0203 | RT | 421 |
| KLGKAGYVT | A0203 | RT | 451 |
| ALQDSGLEV | A0203 | RT | 485 |
| AIIRILQQL | A0203 | Vpr | 59 |
| RILQQLLFI | A0203 | Vpr | 62 |
| VVAIIIAIV | A0203 | Vpu | 13 |
| LWVTVYYGV | A0206 | gp160 | 34 |
| VTVYYGVPV | A0206 | gp160 | 36 |
| NVWATHACV | A0206 | gp160 | 67 |
| QMHEDIISL | A0206 | gp160 | 103 |
| KLTPLCVSL | A0206 | gp160 | 121 |
| KLTSCNTSV | A0206 | gp160 | 192 |
| QRGPGRAFV | A0206 | gp160 | 310 |
| TLKQIASKL | A0206 | gp160 | 341 |
| TMGAASMTL | A0206 | gp160 | 529 |
| AVLSIVNRV | A0206 | gp160 | 700 |
| RLVNGSLAL | A0206 | gp160 | 747 |
| RLRDLLLIV | A0206 | gp160 | 770 |
| LLNATAIAV | A0206 | gp160 | 814 |
| RVIEVVQGA | A0206 | gp160 | 828 |
| RIRQGLERI | A0206 | gp160 | 846 |
| QVRDQAEHL | A0206 | Integrase | 164 |
| LLWKGEGAV | A0206 | Integrase | 241 |
| ATNAACAWL | A0206 | Nef | 50 |
| AAVDLSHFL | A0206 | Nef | 83 |
| YPLTFGWCY | A0206 | Nef | 135 |
| LTFGWCYKL | A0206 | Nef | 137 |
| LEWRFDSRL | A0206 | Nef | 181 |
| AFHHVAREL | A0206 | Nef | 190 |
| SLYNTVATL | A0206 | p17 | 77 |
| TLNAWVKVV | A0206 | p24-p2p7p1 | 19 |
| AMQMLKETI | A0206 | p24-p2p7p1 | 65 |
| AEWDRVHPV | A0206 | p24-p2p7p1 | 78 |
| TLQEQIGWM | A0206 | p24-p2p7p1 | 110 |
| MTNNPPIPV | A0206 | p24-p2p7p1 | 118 |
| RMYSPTSIL | A0206 | p24-p2p7p1 | 143 |
| YVDRFYKTL | A0206 | p24-p2p7p1 | 164 |
| VLAEAMSQV | A0206 | p24-p2p7p1 | 230 |
| LVGPTPVNI | A0206 | Protease | 76 |
| ALVEICTEM | A0206 | RT | 33 |
| YTAFTIPSI | A0206 | RT | 127 |
| VIYQYMDDL | A0206 | RT | 179 |
| YQYMDDLYV | A0206 | RT | 181 |
| KIEELRQHL | A0206 | RT | 201 |
| ILKEPVHGV | A0206 | RT | 309 |
| PLVKLWYQL | A0206 | RT | 421 |
| KLGKAGYVT | A0206 | RT | 451 |
| ALQDSGLEV | A0206 | RT | 485 |
| AIIRILQQL | A0206 | Vpr | 59 |
| RILQQLLFI | A0206 | Vpr | 62 |
| VVAIIIAIV | A0206 | Vpu | 13 |
| LWVTVYYGV | A0211 | gp160 | 34 |
| VTVYYGVPV | A0211 | gp160 | 36 |
| NVWATHACV | A0211 | gp160 | 67 |
| QMHEDIISL | A0211 | gp160 | 103 |
| KLTPLCVSL | A0211 | gp160 | 121 |
| KLTSCNTSV | A0211 | gp160 | 192 |
| QRGPGRAFV | A0211 | gp160 | 310 |
| TLKQIASKL | A0211 | gp160 | 341 |
| TMGAASMTL | A0211 | gp160 | 529 |
| AVLSIVNRV | A0211 | gp160 | 700 |
| RLVNGSLAL | A0211 | gp160 | 747 |
| RLRDLLLIV | A0211 | gp160 | 770 |
| LLNATAIAV | A0211 | gp160 | 814 |
| RVIEVVQGA | A0211 | gp160 | 828 |
| RIRQGLERI | A0211 | gp160 | 846 |
| QVRDQAEHL | A0211 | Integrase | 164 |
| LLWKGEGAV | A0211 | Integrase | 241 |
| ATNAACAWL | A0211 | Nef | 50 |
| AAVDLSHFL | A0211 | Nef | 83 |
| YPLTFGWCY | A0211 | Nef | 135 |
| LTFGWCYKL | A0211 | Nef | 137 |
| LEWRFDSRL | A0211 | Nef | 181 |
| AFHHVAREL | A0211 | Nef | 190 |
| SLYNTVATL | A0211 | p17 | 77 |
| TLNAWVKVV | A0211 | p24-p2p7p1 | 19 |
| AMQMLKETI | A0211 | p24-p2p7p1 | 65 |
| AEWDRVHPV | A0211 | p24-p2p7p1 | 78 |
| TLQEQIGWM | A0211 | p24-p2p7p1 | 110 |
| MTNNPPIPV | A0211 | p24-p2p7p1 | 118 |
| RMYSPTSIL | A0211 | p24-p2p7p1 | 143 |
| YVDRFYKTL | A0211 | p24-p2p7p1 | 164 |
| VLAEAMSQV | A0211 | p24-p2p7p1 | 230 |
| LVGPTPVNI | A0211 | Protease | 76 |
| ALVEICTEM | A0211 | RT | 33 |
| YTAFTIPSI | A0211 | RT | 127 |
| VIYQYMDDL | A0211 | RT | 179 |
| YQYMDDLYV | A0211 | RT | 181 |
| KIEELRQHL | A0211 | RT | 201 |
| ILKEPVHGV | A0211 | RT | 309 |
| PLVKLWYQL | A0211 | RT | 421 |
| KLGKAGYVT | A0211 | RT | 451 |
| ALQDSGLEV | A0211 | RT | 485 |
| AIIRILQQL | A0211 | Vpr | 59 |
| RILQQLLFI | A0211 | Vpr | 62 |
| VVAIIIAIV | A0211 | Vpu | 13 |
| LWVTVYYGV | A0212 | gp160 | 34 |
| VTVYYGVPV | A0212 | gp160 | 36 |
| NVWATHACV | A0212 | gp160 | 67 |
| QMHEDIISL | A0212 | gp160 | 103 |
| KLTPLCVSL | A0212 | gp160 | 121 |
| KLTSCNTSV | A0212 | gp160 | 192 |
| QRGPGRAFV | A0212 | gp160 | 310 |
| TLKQIASKL | A0212 | gp160 | 341 |
| TMGAASMTL | A0212 | gp160 | 529 |
| AVLSIVNRV | A0212 | gp160 | 700 |
| RLVNGSLAL | A0212 | gp160 | 747 |
| RLRDLLLIV | A0212 | gp160 | 770 |
| LLNATAIAV | A0212 | gp160 | 814 |
| RVIEVVQGA | A0212 | gp160 | 828 |
| RIRQGLERI | A0212 | gp160 | 846 |
| QVRDQAEHL | A0212 | Integrase | 164 |
| LLWKGEGAV | A0212 | Integrase | 241 |
| ATNAACAWL | A0212 | Nef | 50 |
| AAVDLSHFL | A0212 | Nef | 83 |
| YPLTFGWCY | A0212 | Nef | 135 |
| LTFGWCYKL | A0212 | Nef | 137 |
| LEWRFDSRL | A0212 | Nef | 181 |
| AFHHVAREL | A0212 | Nef | 190 |
| SLYNTVATL | A0212 | p17 | 77 |
| TLNAWVKVV | A0212 | p24-p2p7p1 | 19 |
| AMQMLKETI | A0212 | p24-p2p7p1 | 65 |
| AEWDRVHPV | A0212 | p24-p2p7p1 | 78 |
| TLQEQIGWM | A0212 | p24-p2p7p1 | 110 |
| MTNNPPIPV | A0212 | p24-p2p7p1 | 118 |
| RMYSPTSIL | A0212 | p24-p2p7p1 | 143 |
| YVDRFYKTL | A0212 | p24-p2p7p1 | 164 |
| VLAEAMSQV | A0212 | p24-p2p7p1 | 230 |
| LVGPTPVNI | A0212 | Protease | 76 |
| ALVEICTEM | A0212 | RT | 33 |
| YTAFTIPSI | A0212 | RT | 127 |
| VIYQYMDDL | A0212 | RT | 179 |
| YQYMDDLYV | A0212 | RT | 181 |
| KIEELRQHL | A0212 | RT | 201 |
| ILKEPVHGV | A0212 | RT | 309 |
| PLVKLWYQL | A0212 | RT | 421 |
| KLGKAGYVT | A0212 | RT | 451 |
| ALQDSGLEV | A0212 | RT | 485 |
| AIIRILQQL | A0212 | Vpr | 59 |
| RILQQLLFI | A0212 | Vpr | 62 |
| VVAIIIAIV | A0212 | Vpu | 13 |
| LWVTVYYGV | A0216 | gp160 | 34 |
| VTVYYGVPV | A0216 | gp160 | 36 |
| NVWATHACV | A0216 | gp160 | 67 |
| QMHEDIISL | A0216 | gp160 | 103 |
| KLTPLCVSL | A0216 | gp160 | 121 |
| KLTSCNTSV | A0216 | gp160 | 192 |
| QRGPGRAFV | A0216 | gp160 | 310 |
| TLKQIASKL | A0216 | gp160 | 341 |
| TMGAASMTL | A0216 | gp160 | 529 |
| AVLSIVNRV | A0216 | gp160 | 700 |
| RLVNGSLAL | A0216 | gp160 | 747 |
| RLRDLLLIV | A0216 | gp160 | 770 |
| LLNATAIAV | A0216 | gp160 | 814 |
| RVIEVVQGA | A0216 | gp160 | 828 |
| RIRQGLERI | A0216 | gp160 | 846 |
| QVRDQAEHL | A0216 | Integrase | 164 |
| LLWKGEGAV | A0216 | Integrase | 241 |
| ATNAACAWL | A0216 | Nef | 50 |
| AAVDLSHFL | A0216 | Nef | 83 |
| YPLTFGWCY | A0216 | Nef | 135 |
| LTFGWCYKL | A0216 | Nef | 137 |
| LEWRFDSRL | A0216 | Nef | 181 |
| AFHHVAREL | A0216 | Nef | 190 |
| SLYNTVATL | A0216 | p17 | 77 |
| TLNAWVKVV | A0216 | p24-p2p7p1 | 19 |
| AMQMLKETI | A0216 | p24-p2p7p1 | 65 |
| AEWDRVHPV | A0216 | p24-p2p7p1 | 78 |
| TLQEQIGWM | A0216 | p24-p2p7p1 | 110 |
| MTNNPPIPV | A0216 | p24-p2p7p1 | 118 |
| RMYSPTSIL | A0216 | p24-p2p7p1 | 143 |
| YVDRFYKTL | A0216 | p24-p2p7p1 | 164 |
| VLAEAMSQV | A0216 | p24-p2p7p1 | 230 |
| LVGPTPVNI | A0216 | Protease | 76 |
| ALVEICTEM | A0216 | RT | 33 |
| YTAFTIPSI | A0216 | RT | 127 |
| VIYQYMDDL | A0216 | RT | 179 |
| YQYMDDLYV | A0216 | RT | 181 |
| KIEELRQHL | A0216 | RT | 201 |
| ILKEPVHGV | A0216 | RT | 309 |
| PLVKLWYQL | A0216 | RT | 421 |
| KLGKAGYVT | A0216 | RT | 451 |
| ALQDSGLEV | A0216 | RT | 485 |
| AIIRILQQL | A0216 | Vpr | 59 |
| RILQQLLFI | A0216 | Vpr | 62 |
| VVAIIIAIV | A0216 | Vpu | 13 |
| LWVTVYYGV | A0219 | gp160 | 34 |
| VTVYYGVPV | A0219 | gp160 | 36 |
| NVWATHACV | A0219 | gp160 | 67 |
| QMHEDIISL | A0219 | gp160 | 103 |
| KLTPLCVSL | A0219 | gp160 | 121 |
| KLTSCNTSV | A0219 | gp160 | 192 |
| QRGPGRAFV | A0219 | gp160 | 310 |
| TLKQIASKL | A0219 | gp160 | 341 |
| TMGAASMTL | A0219 | gp160 | 529 |
| AVLSIVNRV | A0219 | gp160 | 700 |
| RLVNGSLAL | A0219 | gp160 | 747 |
| RLRDLLLIV | A0219 | gp160 | 770 |
| LLNATAIAV | A0219 | gp160 | 814 |
| RVIEVVQGA | A0219 | gp160 | 828 |
| RIRQGLERI | A0219 | gp160 | 846 |
| QVRDQAEHL | A0219 | Integrase | 164 |
| LLWKGEGAV | A0219 | Integrase | 241 |
| ATNAACAWL | A0219 | Nef | 50 |
| AAVDLSHFL | A0219 | Nef | 83 |
| YPLTFGWCY | A0219 | Nef | 135 |
| LTFGWCYKL | A0219 | Nef | 137 |
| LEWRFDSRL | A0219 | Nef | 181 |
| AFHHVAREL | A0219 | Nef | 190 |
| SLYNTVATL | A0219 | p17 | 77 |
| TLNAWVKVV | A0219 | p24-p2p7p1 | 19 |
| AMQMLKETI | A0219 | p24-p2p7p1 | 65 |
| AEWDRVHPV | A0219 | p24-p2p7p1 | 78 |
| TLQEQIGWM | A0219 | p24-p2p7p1 | 110 |
| MTNNPPIPV | A0219 | p24-p2p7p1 | 118 |
| RMYSPTSIL | A0219 | p24-p2p7p1 | 143 |
| YVDRFYKTL | A0219 | p24-p2p7p1 | 164 |
| VLAEAMSQV | A0219 | p24-p2p7p1 | 230 |
| LVGPTPVNI | A0219 | Protease | 76 |
| ALVEICTEM | A0219 | RT | 33 |
| YTAFTIPSI | A0219 | RT | 127 |
| VIYQYMDDL | A0219 | RT | 179 |
| YQYMDDLYV | A0219 | RT | 181 |
| KIEELRQHL | A0219 | RT | 201 |
| ILKEPVHGV | A0219 | RT | 309 |
| PLVKLWYQL | A0219 | RT | 421 |
| KLGKAGYVT | A0219 | RT | 451 |
| ALQDSGLEV | A0219 | RT | 485 |
| AIIRILQQL | A0219 | Vpr | 59 |
| RILQQLLFI | A0219 | Vpr | 62 |
| VVAIIIAIV | A0219 | Vpu | 13 |
| SLWDQSLKP | A0301 | gp160 | 110 |
| VSFEPIPIH | A0301 | gp160 | 208 |
| HSFNCGGEF | A0301 | gp160 | 374 |
| AVDLSHFLK | A0301 | Nef | 84 |
| DLSHFLKEK | A0301 | Nef | 86 |
| ILDLWIYHT | A0301 | Nef | 109 |
| PLTFGWCYK | A0301 | Nef | 136 |
| AFHHVAREL | A0301 | Nef | 190 |
| KIRLRPGGK | A0301 | p17 | 18 |
| RLRPGGKKK | A0301 | p17 | 20 |
| TVRLIKLLY | A0301 | Rev | 15 |
| KLLYQSNPP | A0301 | Rev | 20 |
| RILGTYLGR | A0301 | Rev | 58 |
| ALVEICTEM | A0301 | RT | 33 |
| NTPVFAIKK | A0301 | RT | 57 |
| GIPHPAGLK | A0301 | RT | 93 |
| AIFQSSMTK | A0301 | RT | 158 |
| QIYPGIKVR | A0301 | RT | 269 |
| QIIEQLIKK | A0301 | RT | 520 |
| KVYLAWVPA | A0301 | RT | 530 |
| TACTNCYCK | A0301 | Tat | 20 |
| HMYVSGKAR | A0301 | Vif | 28 |
| KLTEDRWNK | A0301 | Vif | 168 |
| SVITQACPK | A1101 | gp160 | 199 |
| NTLKQIASK | A1101 | gp160 | 340 |
| AVDLSHFLK | A1101 | Nef | 84 |
| DLSHFLKEK | A1101 | Nef | 86 |
| TLYCVHQRI | A1101 | p17 | 84 |
| AIFQSSMTK | A1101 | RT | 158 |
| QIYPGIKVR | A1101 | RT | 269 |
| FVNTPPLVK | A1101 | RT | 416 |
| QIIEQLIKK | A1101 | RT | 520 |
| RYLKDQQLL | A2301 | gp160 | 585 |
| KYKLKHIVW | A2301 | p17 | 28 |
| IYQEPFKNL | A2301 | RT | 341 |
| IQRGPGRAF | A2402 | gp160 | 309 |
| FYCNSTQLF | A2402 | gp160 | 383 |
| RYLKDQQLL | A2402 | gp160 | 585 |
| WYIKLFIMI | A2402 | gp160 | 680 |
| SYHRLRDLL | A2402 | gp160 | 767 |
| HSQRRQDIL | A2402 | Nef | 102 |
| RQDILDLWI | A2402 | Nef | 106 |
| GYFPDWQNY | A2402 | Nef | 119 |
| DSRLAFHHV | A2402 | Nef | 186 |
| AFHHVAREL | A2402 | Nef | 190 |
| KYKLKHIVW | A2402 | p17 | 28 |
| EIYKRWIIL | A2402 | p24-p2p7p1 | 128 |
| DYVDRFYKT | A2402 | p24-p2p7p1 | 163 |
| VYYDPSKDL | A2402 | RT | 317 |
| IYQEPFKNL | A2402 | RT | 341 |
| IQRGPGRAF | A2403 | gp160 | 309 |
| FYCNSTQLF | A2403 | gp160 | 383 |
| RYLKDQQLL | A2403 | gp160 | 585 |
| WYIKLFIMI | A2403 | gp160 | 680 |
| SYHRLRDLL | A2403 | gp160 | 767 |
| HSQRRQDIL | A2403 | Nef | 102 |
| RQDILDLWI | A2403 | Nef | 106 |
| GYFPDWQNY | A2403 | Nef | 119 |
| DSRLAFHHV | A2403 | Nef | 186 |
| AFHHVAREL | A2403 | Nef | 190 |
| KYKLKHIVW | A2403 | p17 | 28 |
| EIYKRWIIL | A2403 | p24-p2p7p1 | 128 |
| DYVDRFYKT | A2403 | p24-p2p7p1 | 163 |
| VYYDPSKDL | A2403 | RT | 317 |
| IYQEPFKNL | A2403 | RT | 341 |
| EVIPMFSAL | A2601 | p24-p2p7p1 | 35 |
| YVDRFYKTL | A2601 | p24-p2p7p1 | 164 |
| ETKLGKAGY | A2601 | RT | 449 |
| EVIPMFSAL | A2602 | p24-p2p7p1 | 35 |
| YVDRFYKTL | A2602 | p24-p2p7p1 | 164 |
| ETKLGKAGY | A2602 | RT | 449 |
| SFEPIPIHY | A2902 | gp160 | 209 |
| FNCGGEFFY | A2902 | gp160 | 376 |
| RIKQIINMW | A2902 | gp160 | 419 |
| YFPDWQNYT | A2902 | Nef | 120 |
| LYNTVATLY | A2902 | p17 | 78 |
| NCYCKKCCF | A2902 | Tat | 24 |
| HIVSPRCEY | A2902 | Vif | 127 |
| QRGPGRAFV | A3001 | gp160 | 310 |
| IVNRVRQGY | A3001 | gp160 | 704 |
| KYWWNLLQY | A3001 | gp160 | 794 |
| KIQNFRVYY | A3001 | Integrase | 219 |
| RLRPGGKKK | A3001 | p17 | 20 |
| KQNPDIVIY | A3001 | RT | 173 |
| KLNWASQIY | A3001 | RT | 263 |
| QRGPGRAFV | A3002 | gp160 | 310 |
| IVNRVRQGY | A3002 | gp160 | 704 |
| KYWWNLLQY | A3002 | gp160 | 794 |
| KIQNFRVYY | A3002 | Integrase | 219 |
| RLRPGGKKK | A3002 | p17 | 20 |
| KQNPDIVIY | A3002 | RT | 173 |
| KLNWASQIY | A3002 | RT | 263 |
| VRYPLTFGW | A3301 | Nef | 133 |
| RLAFHHVAR | A3301 | Nef | 188 |
| MVHQAISPR | A3301 | p24-p2p7p1 | 10 |
| AIFQSSMTK | A3301 | RT | 158 |
| FYVDGAANR | A3301 | RT | 440 |
| EYRKILRQR | A3301 | Vpu | 29 |
| ETAYFLLKL | A6801 | Integrase | 96 |
| VTLWQRPLV | A6801 | Protease | 3 |
| DTVLEEMSL | A6801 | Protease | 30 |
| NTPVFAIKK | A6801 | RT | 57 |
| AIFQSSMTK | A6801 | RT | 158 |
| ETAYFLLKL | A6802 | Integrase | 96 |
| VTLWQRPLV | A6802 | Protease | 3 |
| DTVLEEMSL | A6802 | Protease | 30 |
| NTPVFAIKK | A6802 | RT | 57 |
| AIFQSSMTK | A6802 | RT | 158 |
| RAEPAADRV | A6901 | Nef | 22 |
| RVGAASRDL | A6901 | Nef | 29 |
| IPRRIRQGL | B0702 | gp160 | 843 |
| LPPVVAKEI | B0702 | Integrase | 28 |
| FPVTPQVPL | B0702 | Nef | 68 |
| TPQVPLRPM | B0702 | Nef | 71 |
| RPMTYKAAV | B0702 | Nef | 77 |
| TPGPGVRYP | B0702 | Nef | 128 |
| YPLTFGWCY | B0702 | Nef | 135 |
| KIRLRPGGK | B0702 | p17 | 18 |
| SPRTLNAWV | B0702 | p24-p2p7p1 | 16 |
| ATPQDLNTM | B0702 | p24-p2p7p1 | 47 |
| TPQDLNTML | B0702 | p24-p2p7p1 | 48 |
| HPVHAGPIA | B0702 | p24-p2p7p1 | 84 |
| ANPDCKTIL | B0702 | p24-p2p7p1 | 194 |
| GPGHKARVL | B0702 | p24-p2p7p1 | 223 |
| YPLTSLRSL | B0702 | p24-p2p7p1 | 352 |
| SPAIFQSSM | B0702 | RT | 156 |
| IPLTEEAEL | B0702 | RT | 293 |
| YLAWVPAHK | B0702 | RT | 532 |
| FPRIWLHGL | B0702 | Vpr | 34 |
| RVKEKYQHL | B0801 | gp160 | 2 |
| FNCGGEFFY | B0801 | gp160 | 376 |
| GGKKKYKLK | B0801 | p17 | 24 |
| ELRSLYNTV | B0801 | p17 | 74 |
| EIKDTKEAL | B0801 | p17 | 93 |
| GEIYKRWII | B0801 | p24-p2p7p1 | 127 |
| NANPDCKTI | B0801 | p24-p2p7p1 | 193 |
| DCKTILKAL | B0801 | p24-p2p7p1 | 197 |
| GPKVKQWPL | B0801 | RT | 18 |
| RVKEKYQHL | B0802 | gp160 | 2 |
| FNCGGEFFY | B0802 | gp160 | 376 |
| GGKKKYKLK | B0802 | p17 | 24 |
| ELRSLYNTV | B0802 | p17 | 74 |
| EIKDTKEAL | B0802 | p17 | 93 |
| GEIYKRWII | B0802 | p24-p2p7p1 | 127 |
| NANPDCKTI | B0802 | p24-p2p7p1 | 193 |
| DCKTILKAL | B0802 | p24-p2p7p1 | 197 |
| GPKVKQWPL | B0802 | RT | 18 |
| SFNCGGEFF | B1501 | gp160 | 375 |
| RAIEAQQHL | B1501 | gp160 | 557 |
| THLEGKVIL | B1501 | Integrase | 66 |
| IKQEFGIPY | B1501 | Integrase | 135 |
| RKAKIIRDY | B1501 | Integrase | 263 |
| RMRRAEPAA | B1501 | Nef | 19 |
| MTYKAAVDL | B1501 | Nef | 79 |
| AAVDLSHFL | B1501 | Nef | 83 |
| YFPDWQNYT | B1501 | Nef | 120 |
| LTFGWCYKL | B1501 | Nef | 137 |
| WRFDSRLAF | B1501 | Nef | 183 |
| RLRPGGKKK | B1501 | p17 | 20 |
| RFAVNPGLL | B1501 | p17 | 43 |
| VKVVEEKAF | B1501 | p24-p2p7p1 | 24 |
| FSPEVIPMF | B1501 | p24-p2p7p1 | 32 |
| GHQAAMQML | B1501 | p24-p2p7p1 | 61 |
| GLNKIVRMY | B1501 | p24-p2p7p1 | 137 |
| YVDRFYKTL | B1501 | p24-p2p7p1 | 164 |
| GHKAIGTVL | B1501 | Protease | 68 |
| IHSISERIL | B1501 | Rev | 52 |
| IPLTEEAEL | B1501 | RT | 293 |
| DVKQLTEAV | B1501 | RT | 364 |
| ITKALGISY | B1501 | Tat | 39 |
| WHLGQGVSI | B1501 | Vif | 79 |
| AVRHFPRIW | B1501 | Vpr | 30 |
| TEKLWVTVY | B1801 | gp160 | 31 |
| YDTEVHNVW | B1801 | gp160 | 61 |
| QDILDLWIY | B1801 | Nef | 107 |
| YPLTFGWCY | B1801 | Nef | 135 |
| NNETPGIRY | B1801 | RT | 136 |
| NPDIVIYQY | B1801 | RT | 175 |
| GRAFVTIGK | B2705 | gp160 | 314 |
| GRRGWEALK | B2705 | gp160 | 786 |
| KIRLRPGGK | B2705 | p17 | 18 |
| IRLRPGGKK | B2705 | p17 | 19 |
| RWIILGLNK | B2705 | p24-p2p7p1 | 132 |
| VRHFPRIWL | B2705 | Vpr | 31 |
| DPNPQEVVL | B3501 | gp160 | 78 |
| RPVVSTQLL | B3501 | gp160 | 252 |
| TAVPWNASW | B3501 | gp160 | 606 |
| FPVTPQVPL | B3501 | Nef | 68 |
| WIYHTQGYF | B3501 | Nef | 113 |
| YPLTFGWCY | B3501 | Nef | 135 |
| RPGGKKKYK | B3501 | p17 | 22 |
| WASRELERF | B3501 | p17 | 36 |
| HSNQVSQNY | B3501 | p17 | 124 |
| PPIPVGEIY | B3501 | p24-p2p7p1 | 122 |
| TVLDVGDAY | B3501 | RT | 107 |
| FSVPLDEDF | B3501 | RT | 116 |
| SPAIFQSSM | B3501 | RT | 156 |
| NPDIVIYQY | B3501 | RT | 175 |
| IPLTEEAEL | B3501 | RT | 293 |
| EPIVGAETF | B3501 | RT | 432 |
| DARLVITTY | B3501 | Vif | 61 |
| HTGERDWHL | B3501 | Vif | 73 |
| TPQDLNTML | B3901 | p24-p2p7p1 | 48 |
| HPVHAGPIA | B3901 | p24-p2p7p1 | 84 |
| LEKHGAITS | B4001 | Nef | 37 |
| AAVDLSHFL | B4001 | Nef | 83 |
| KEKGGLEGL | B4001 | Nef | 92 |
| GELDRWEKI | B4001 | p17 | 11 |
| SEGATPQDL | B4001 | p24-p2p7p1 | 44 |
| KETINEEAA | B4001 | p24-p2p7p1 | 70 |
| EEAAEWDRV | B4001 | p24-p2p7p1 | 75 |
| AEWDRVHPV | B4001 | p24-p2p7p1 | 78 |
| CTERQANFL | B4001 | p24-p2p7p1 | 294 |
| KELYPLTSL | B4001 | p24-p2p7p1 | 349 |
| IEELRQHLL | B4001 | RT | 202 |
| REPHNEWTL | B4001 | Vpr | 12 |
| LEKHGAITS | B4002 | Nef | 37 |
| AAVDLSHFL | B4002 | Nef | 83 |
| KEKGGLEGL | B4002 | Nef | 92 |
| GELDRWEKI | B4002 | p17 | 11 |
| SEGATPQDL | B4002 | p24-p2p7p1 | 44 |
| KETINEEAA | B4002 | p24-p2p7p1 | 70 |
| EEAAEWDRV | B4002 | p24-p2p7p1 | 75 |
| AEWDRVHPV | B4002 | p24-p2p7p1 | 78 |
| CTERQANFL | B4002 | p24-p2p7p1 | 294 |
| KELYPLTSL | B4002 | p24-p2p7p1 | 349 |
| IEELRQHLL | B4002 | RT | 202 |
| REPHNEWTL | B4002 | Vpr | 12 |
| TEKLWVTVY | B4402 | gp160 | 31 |
| LYNTVATLY | B4402 | p17 | 78 |
| EEKAFSPEV | B4402 | p24-p2p7p1 | 28 |
| SEGATPQDL | B4402 | p24-p2p7p1 | 44 |
| QEPIDKELY | B4402 | p24-p2p7p1 | 344 |
| EEMSLPGRW | B4402 | Protease | 34 |
| EELRQHLLR | B4402 | RT | 203 |
| TEKLWVTVY | B4403 | gp160 | 31 |
| LYNTVATLY | B4403 | p17 | 78 |
| EEKAFSPEV | B4403 | p24-p2p7p1 | 28 |
| SEGATPQDL | B4403 | p24-p2p7p1 | 44 |
| QEPIDKELY | B4403 | p24-p2p7p1 | 344 |
| EEMSLPGRW | B4403 | Protease | 34 |
| EELRQHLLR | B4403 | RT | 203 |
| EEKAFSPEV | B4501 | p24-p2p7p1 | 28 |
| AETFYVDGA | B4501 | RT | 437 |
| DPNPQEVVL | B5101 | gp160 | 78 |
| LPCRIKQII | B5101 | gp160 | 416 |
| RAIEAQQHL | B5101 | gp160 | 557 |
| GACRAIRHI | B5101 | gp160 | 835 |
| LPPVVAKEI | B5101 | Integrase | 28 |
| YFPDWQNYT | B5101 | Nef | 120 |
| DSRLAFHHV | B5101 | Nef | 186 |
| AFHHVAREL | B5101 | Nef | 190 |
| NANPDCKTI | B5101 | p24-p2p7p1 | 193 |
| EKEGKISKI | B5101 | RT | 42 |
| QGWKGSPAI | B5101 | RT | 151 |
| IPLTEEAEL | B5101 | RT | 293 |
| EPIVGAETF | B5101 | RT | 432 |
| EAVRHFPRI | B5101 | Vpr | 29 |
| YPLTFGWCY | B5301 | Nef | 135 |
| TPQDLNTML | B5301 | p24-p2p7p1 | 48 |
| PPIPVGEIY | B5301 | p24-p2p7p1 | 122 |
| QASQEVKNW | B5301 | p24-p2p7p1 | 176 |
| ASQEVKNWM | B5301 | p24-p2p7p1 | 177 |
| TPPQKQEPI | B5301 | p24-p2p7p1 | 339 |
| YPLTSLRSL | B5301 | p24-p2p7p1 | 352 |
| IPLTEEAEL | B5301 | RT | 293 |
| RAIEAQQHL | B5701 | gp160 | 557 |
| KTAVQMAVF | B5701 | Integrase | 173 |
| KAAVDLSHF | B5701 | Nef | 82 |
| HTQGYFPDW | B5701 | Nef | 116 |
| YFPDWQNYT | B5701 | Nef | 120 |
| YTPGPGVRY | B5701 | Nef | 127 |
| LTFGWCYKL | B5701 | Nef | 137 |
| ISPRTLNAW | B5701 | p24-p2p7p1 | 15 |
| FSPEVIPMF | B5701 | p24-p2p7p1 | 32 |
| STLQEQIGW | B5701 | p24-p2p7p1 | 109 |
| QASQEVKNW | B5701 | p24-p2p7p1 | 176 |
| FSVPLDEDF | B5701 | RT | 116 |
| IVLPEKDSW | B5701 | RT | 244 |
| ITTESIVIW | B5701 | RT | 375 |
| VSGKARGWF | B5701 | Vif | 31 |
| AVRHFPRIW | B5701 | Vpr | 30 |
| RAIEAQQHL | B5801 | gp160 | 557 |
| KTAVQMAVF | B5801 | Integrase | 173 |
| KAAVDLSHF | B5801 | Nef | 82 |
| HTQGYFPDW | B5801 | Nef | 116 |
| YTPGPGVRY | B5801 | Nef | 127 |
| ISPRTLNAW | B5801 | p24-p2p7p1 | 15 |
| FSPEVIPMF | B5801 | p24-p2p7p1 | 32 |
| STLQEQIGW | B5801 | p24-p2p7p1 | 109 |
| QASQEVKNW | B5801 | p24-p2p7p1 | 176 |
| IVLPEKDSW | B5801 | RT | 244 |
| ITTESIVIW | B5801 | RT | 375 |
| VSGKARGWF | B5801 | Vif | 31 |
| AVRHFPRIW | B5801 | Vpr | 30 |
