# Supplementary material for: T-Cell Epitope Prediction: Rescaling Can Mask Biological Variation between MHC Molecules
Source: PLoS Comput Biol. 2009 Mar 20;5(3):e1000327. doi: 10.1371/journal.pcbi.1000327 (PMC2650421; doi:10.1371/journal.pcbi.1000327)
Supplement: Dataset S4 — The Lanl 179 dataset. (0.16 MB DOC) [file pcbi.1000327.s004.doc]

**Lanl^179^**

Dataset S4: The Lanl^179^ dataset

| epitope | allele | protein | HXB2 start |
| --- | --- | --- | --- |
| RRGWEALKY | A1 | gp160 | 787 |
| WIYHTQGYF | A1 | Nef | 113 |
| YFPDWQNYT | A1 | Nef | 120 |
| GSEELRSLY | A1 | p17 | 71 |
| QRPLVTIKI | A1 | Protease | 7 |
| ISERILGTY | A1 | Rev | 55 |
| LWVTVYYGV | A2 | gp160 | 34 |
| VTVYYGVPV | A2 | gp160 | 36 |
| NVWATHACV | A2 | gp160 | 67 |
| QMHEDIISL | A2 | gp160 | 103 |
| KLTPLCVSL | A2 | gp160 | 121 |
| KLTSCNTSV | A2 | gp160 | 192 |
| QRGPGRAFV | A2 | gp160 | 310 |
| TLKQIASKL | A2 | gp160 | 341 |
| TMGAASMTL | A2 | gp160 | 529 |
| AVLSIVNRV | A2 | gp160 | 700 |
| RLVNGSLAL | A2 | gp160 | 747 |
| RLRDLLLIV | A2 | gp160 | 770 |
| LLNATAIAV | A2 | gp160 | 814 |
| RVIEVVQGA | A2 | gp160 | 828 |
| RIRQGLERI | A2 | gp160 | 846 |
| QVRDQAEHL | A2 | Integrase | 164 |
| LLWKGEGAV | A2 | Integrase | 241 |
| ATNAACAWL | A2 | Nef | 50 |
| AAVDLSHFL | A2 | Nef | 83 |
| YPLTFGWCY | A2 | Nef | 135 |
| LTFGWCYKL | A2 | Nef | 137 |
| LEWRFDSRL | A2 | Nef | 181 |
| AFHHVAREL | A2 | Nef | 190 |
| SLYNTVATL | A2 | p17 | 77 |
| TLNAWVKVV | A2 | p24-p2p7p1 | 19 |
| AMQMLKETI | A2 | p24-p2p7p1 | 65 |
| AEWDRVHPV | A2 | p24-p2p7p1 | 78 |
| TLQEQIGWM | A2 | p24-p2p7p1 | 110 |
| MTNNPPIPV | A2 | p24-p2p7p1 | 118 |
| RMYSPTSIL | A2 | p24-p2p7p1 | 143 |
| YVDRFYKTL | A2 | p24-p2p7p1 | 164 |
| VLAEAMSQV | A2 | p24-p2p7p1 | 230 |
| LVGPTPVNI | A2 | Protease | 76 |
| ALVEICTEM | A2 | RT | 33 |
| YTAFTIPSI | A2 | RT | 127 |
| VIYQYMDDL | A2 | RT | 179 |
| YQYMDDLYV | A2 | RT | 181 |
| KIEELRQHL | A2 | RT | 201 |
| ILKEPVHGV | A2 | RT | 309 |
| PLVKLWYQL | A2 | RT | 421 |
| KLGKAGYVT | A2 | RT | 451 |
| ALQDSGLEV | A2 | RT | 485 |
| AIIRILQQL | A2 | Vpr | 59 |
| RILQQLLFI | A2 | Vpr | 62 |
| VVAIIIAIV | A2 | Vpu | 13 |
| SLWDQSLKP | A3 | gp160 | 110 |
| VSFEPIPIH | A3 | gp160 | 208 |
| HSFNCGGEF | A3 | gp160 | 374 |
| AVDLSHFLK | A3 | Nef | 84 |
| DLSHFLKEK | A3 | Nef | 86 |
| ILDLWIYHT | A3 | Nef | 109 |
| PLTFGWCYK | A3 | Nef | 136 |
| AFHHVAREL | A3 | Nef | 190 |
| KIRLRPGGK | A3 | p17 | 18 |
| RLRPGGKKK | A3 | p17 | 20 |
| TVRLIKLLY | A3 | Rev | 15 |
| KLLYQSNPP | A3 | Rev | 20 |
| RILGTYLGR | A3 | Rev | 58 |
| ALVEICTEM | A3 | RT | 33 |
| NTPVFAIKK | A3 | RT | 57 |
| GIPHPAGLK | A3 | RT | 93 |
| AIFQSSMTK | A3 | RT | 158 |
| QIYPGIKVR | A3 | RT | 269 |
| QIIEQLIKK | A3 | RT | 520 |
| KVYLAWVPA | A3 | RT | 530 |
| TACTNCYCK | A3 | Tat | 20 |
| HMYVSGKAR | A3 | Vif | 28 |
| KLTEDRWNK | A3 | Vif | 168 |
| IQRGPGRAF | A24 | gp160 | 309 |
| FYCNSTQLF | A24 | gp160 | 383 |
| RYLKDQQLL | A24 | gp160 | 585 |
| WYIKLFIMI | A24 | gp160 | 680 |
| SYHRLRDLL | A24 | gp160 | 767 |
| HSQRRQDIL | A24 | Nef | 102 |
| RQDILDLWI | A24 | Nef | 106 |
| GYFPDWQNY | A24 | Nef | 119 |
| DSRLAFHHV | A24 | Nef | 186 |
| AFHHVAREL | A24 | Nef | 190 |
| KYKLKHIVW | A24 | p17 | 28 |
| EIYKRWIIL | A24 | p24-p2p7p1 | 128 |
| DYVDRFYKT | A24 | p24-p2p7p1 | 163 |
| VYYDPSKDL | A24 | RT | 317 |
| IYQEPFKNL | A24 | RT | 341 |
| EVIPMFSAL | A26 | p24-p2p7p1 | 35 |
| YVDRFYKTL | A26 | p24-p2p7p1 | 164 |
| ETKLGKAGY | A26 | RT | 449 |
| IPRRIRQGL | B7 | gp160 | 843 |
| LPPVVAKEI | B7 | Integrase | 28 |
| FPVTPQVPL | B7 | Nef | 68 |
| TPQVPLRPM | B7 | Nef | 71 |
| RPMTYKAAV | B7 | Nef | 77 |
| TPGPGVRYP | B7 | Nef | 128 |
| YPLTFGWCY | B7 | Nef | 135 |
| KIRLRPGGK | B7 | p17 | 18 |
| SPRTLNAWV | B7 | p24-p2p7p1 | 16 |
| ATPQDLNTM | B7 | p24-p2p7p1 | 47 |
| TPQDLNTML | B7 | p24-p2p7p1 | 48 |
| HPVHAGPIA | B7 | p24-p2p7p1 | 84 |
| ANPDCKTIL | B7 | p24-p2p7p1 | 194 |
| GPGHKARVL | B7 | p24-p2p7p1 | 223 |
| YPLTSLRSL | B7 | p24-p2p7p1 | 352 |
| SPAIFQSSM | B7 | RT | 156 |
| IPLTEEAEL | B7 | RT | 293 |
| YLAWVPAHK | B7 | RT | 532 |
| FPRIWLHGL | B7 | Vpr | 34 |
| RVKEKYQHL | B8 | gp160 | 2 |
| FNCGGEFFY | B8 | gp160 | 376 |
| GGKKKYKLK | B8 | p17 | 24 |
| ELRSLYNTV | B8 | p17 | 74 |
| EIKDTKEAL | B8 | p17 | 93 |
| GEIYKRWII | B8 | p24-p2p7p1 | 127 |
| NANPDCKTI | B8 | p24-p2p7p1 | 193 |
| DCKTILKAL | B8 | p24-p2p7p1 | 197 |
| GPKVKQWPL | B8 | RT | 18 |
| GRAFVTIGK | B27 | gp160 | 314 |
| GRRGWEALK | B27 | gp160 | 786 |
| KIRLRPGGK | B27 | p17 | 18 |
| IRLRPGGKK | B27 | p17 | 19 |
| KRWIILGLN | B27 | p24-p2p7p1 | 131 |
| RWIILGLNK | B27 | p24-p2p7p1 | 132 |
| VRHFPRIWL | B27 | Vpr | 31 |
| TPQDLNTML | B39 | p24-p2p7p1 | 48 |
| HPVHAGPIA | B39 | p24-p2p7p1 | 84 |
| LEKHGAITS | B44 | Nef | 37 |
| AAVDLSHFL | B44 | Nef | 83 |
| KEKGGLEGL | B44 | Nef | 92 |
| GELDRWEKI | B44 | p17 | 11 |
| SEGATPQDL | B44 | p24-p2p7p1 | 44 |
| KETINEEAA | B44 | p24-p2p7p1 | 70 |
| EEAAEWDRV | B44 | p24-p2p7p1 | 75 |
| AEWDRVHPV | B44 | p24-p2p7p1 | 78 |
| CTERQANFL | B44 | p24-p2p7p1 | 294 |
| KELYPLTSL | B44 | p24-p2p7p1 | 349 |
| IEELRQHLL | B44 | RT | 202 |
| REPHNEWTL | B44 | Vpr | 12 |
| RAIEAQQHL | B58 | gp160 | 557 |
| KTAVQMAVF | B58 | Integrase | 173 |
| KAAVDLSHF | B58 | Nef | 82 |
| HTQGYFPDW | B58 | Nef | 116 |
| YTPGPGVRY | B58 | Nef | 127 |
| ISPRTLNAW | B58 | p24-p2p7p1 | 15 |
| FSPEVIPMF | B58 | p24-p2p7p1 | 32 |
| STLQEQIGW | B58 | p24-p2p7p1 | 109 |
| QASQEVKNW | B58 | p24-p2p7p1 | 176 |
| IVLPEKDSW | B58 | RT | 244 |
| ITTESIVIW | B58 | RT | 375 |
| VSGKARGWF | B58 | Vif | 31 |
| AVRHFPRIW | B58 | Vpr | 30 |
| SFNCGGEFF | B62 | gp160 | 375 |
| RAIEAQQHL | B62 | gp160 | 557 |
| THLEGKVIL | B62 | Integrase | 66 |
| IKQEFGIPY | B62 | Integrase | 135 |
| RKAKIIRDY | B62 | Integrase | 263 |
| RMRRAEPAA | B62 | Nef | 19 |
| MTYKAAVDL | B62 | Nef | 79 |
| AAVDLSHFL | B62 | Nef | 83 |
| YFPDWQNYT | B62 | Nef | 120 |
| LTFGWCYKL | B62 | Nef | 137 |
| WRFDSRLAF | B62 | Nef | 183 |
| RLRPGGKKK | B62 | p17 | 20 |
| RFAVNPGLL | B62 | p17 | 43 |
| VKVVEEKAF | B62 | p24-p2p7p1 | 24 |
| FSPEVIPMF | B62 | p24-p2p7p1 | 32 |
| GHQAAMQML | B62 | p24-p2p7p1 | 61 |
| GLNKIVRMY | B62 | p24-p2p7p1 | 137 |
| YVDRFYKTL | B62 | p24-p2p7p1 | 164 |
| GHKAIGTVL | B62 | Protease | 68 |
| IHSISERIL | B62 | Rev | 52 |
| IPLTEEAEL | B62 | RT | 293 |
| DVKQLTEAV | B62 | RT | 364 |
| ITKALGISY | B62 | Tat | 39 |
| WHLGQGVSI | B62 | Vif | 79 |
| AVRHFPRIW | B62 | Vpr | 30 |
