# Supplementary material for: T-Cell Epitope Prediction: Rescaling Can Mask Biological Variation between MHC Molecules
Source: PLoS Comput Biol. 2009 Mar 20;5(3):e1000327. doi: 10.1371/journal.pcbi.1000327 (PMC2650421; doi:10.1371/journal.pcbi.1000327)
Supplement: Table S1 — The specificity of non-rescaled and rescaled results at specified sensitivity values. (0.03 MB DOC) [file pcbi.1000327.s005.doc]

Table S1: The specificity of non-rescaled and rescaled results at specified sensitivity values. Epitope datasets taken from [1].

| Sensitivity | No Rescaling | Rescaling | Epitope Set |
| --- | --- | --- | --- |
| 0.3 | 0.995 | 0.989 | HIV^216^ |
| 0.6 | 0.987 | 0.977 |
| 0.8 | 0.921 | 0.891 |
| 0.3 | 0.998 | 0.997 | SYF^863^ |
| 0.6 | 0.991 | 0.991 |
| 0.8 | 0.974 | 0.973 |

[1] Mette Larsen, Claus Lundegaard, Kasper Lamberth, Soren Buus, Ole Lund, and Morten Nielsen. Large-Scale validation of methods for cytotoxic T-lymphocyte epitope prediction. *BMC Bioinformatics*, 8(1):424, Oct 2007.
