# Supplementary material for: T-Cell Epitope Prediction: Rescaling Can Mask Biological Variation between MHC Molecules
Source: PLoS Comput Biol. 2009 Mar 20;5(3):e1000327. doi: 10.1371/journal.pcbi.1000327 (PMC2650421; doi:10.1371/journal.pcbi.1000327)
Supplement: Table S2 — The fraction of the total number of epitopes in the 2 epitope datasets among the top 5% of predicted binding affinities. (0.03 MB DOC) [file pcbi.1000327.s006.doc]

Table S2: The fraction of the total number of epitopes in the 2 epitope datasets among the top 5% of predicted binding affinities.

| Epitope Set | No Rescaling | Rescaling |
| --- | --- | --- |
| SYF^863^ | 0.885 | 0.877 |
| HIV^216^ | 0.718 | 0.690 |
