# Supplementary material for: T-Cell Epitope Prediction: Rescaling Can Mask Biological Variation between MHC Molecules
Source: PLoS Comput Biol. 2009 Mar 20;5(3):e1000327. doi: 10.1371/journal.pcbi.1000327 (PMC2650421; doi:10.1371/journal.pcbi.1000327)
Supplement: Figure S1 — The result of the ROC curve analysis, using the Lanl 661 dataset and excluding any alleles (7 in total) that had an AUC<0.9 from figure 2 (bootstrap: p<0.001). (0.03 MB DOC) [file pcbi.1000327.s007.doc]

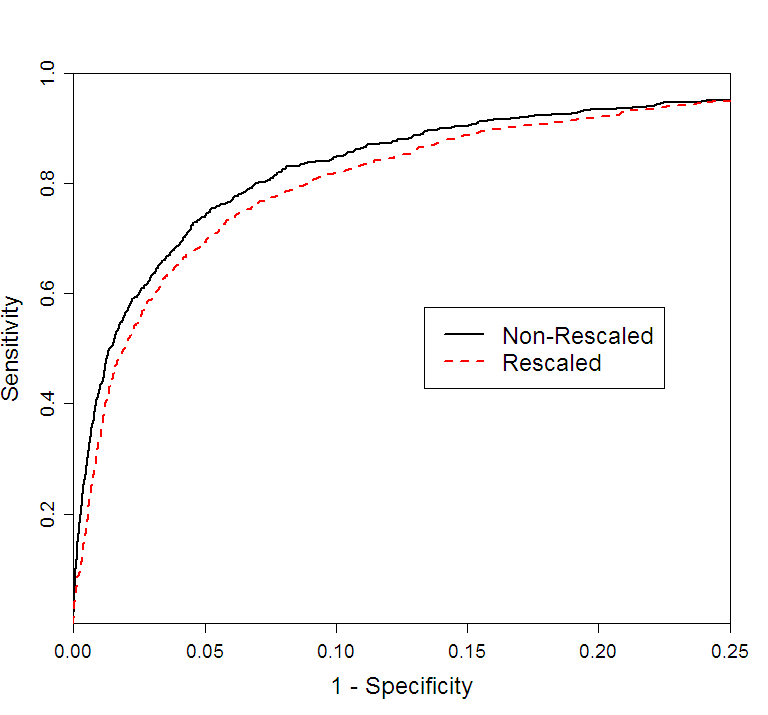


Figure S1: The result of the ROC curve analysis, using the Lanl^661^ dataset and excluding any alleles (7 in total) that had an AUC < 0.9 from figure 2 (bootstrap: p < 0.001).
