# Supplementary material for: T-Cell Epitope Prediction: Rescaling Can Mask Biological Variation between MHC Molecules
Source: PLoS Comput Biol. 2009 Mar 20;5(3):e1000327. doi: 10.1371/journal.pcbi.1000327 (PMC2650421; doi:10.1371/journal.pcbi.1000327)
Supplement: Figure S2 — A comparison of ranks between rescaled and non-rescaled predicted binding affinities. (0.03 MB DOC) [file pcbi.1000327.s008.doc]

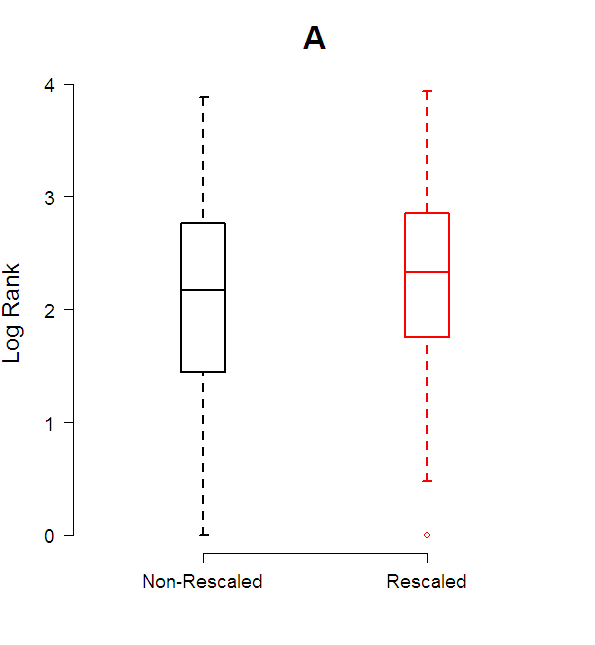

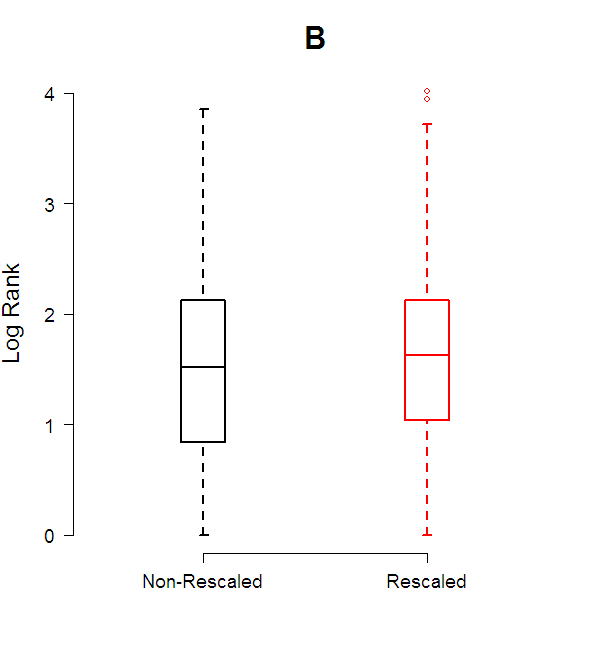


Figure S2: (A) A box plot showing the summary statistics of the ranks (log_10_) of each of the 216 epitopes in the HIV dataset among all overlapping 9-mer in the epitopes’ source proteins. The ranks of the epitopes were significantly lower for non-rescaled scores compared to rescaled scores (Paired Wilcoxon ranked sum test, P < 0.001). The non-rescaled scores produced a higher rank for 170 epitopes and rescaled scores for 24 epitopes. (B) The same analysis using 863 epitopes from the SYFPEITHI dataset. The ranks of the epitopes were significantly lower for non-rescaled scores compared to rescaled scores (Paired Wilcoxon ranked sum test, P < 0.001). The non-rescaled scores produced a higher rank for 474 epitopes and rescaled scores for 369 epitopes.
