# Supplementary material for: T-Cell Epitope Prediction: Rescaling Can Mask Biological Variation between MHC Molecules
Source: PLoS Comput Biol. 2009 Mar 20;5(3):e1000327. doi: 10.1371/journal.pcbi.1000327 (PMC2650421; doi:10.1371/journal.pcbi.1000327)
Supplement: Figure S3 — The relationship between rescaled/non-rescaled predicted binding affinities and experimental binding affinities. (0.19 MB DOC) [file pcbi.1000327.s009.doc]

**The relationship between rescaled / non-rescaled predicted binding affinities and experimental binding affinities**

A set of 128 experimentally-derived epitope-allele binding affinities was extracted from the Immune Epitope Database and Analysis Resource [1]. This set of epitopes was known to have no involvement in the training of any of the allelic predictors in NetMHC v3.0 or NetCTL v1.2.
The relationship between the rescaled / non-rescaled predicted binding affinities and the experimental binding affinities was investigated (figure S3 A). Rescaling resulted in a significantly larger error (the difference between predicted and experimental affinity) compared to predicted binding affinities that were not rescaled (p < 0.001). Although rescaling would naturally result in a larger quantitative error, additionally, the correlation between predicted and experimental affinities was weaker with rescaling than without (rescaled: p < 0.001, Spearman’s R_s_ = 0.40; not rescaled: p < 0.001, Spearman’s R_s_ = 0.51).

The analysis was repeated using a second experimental dataset. This second dataset came from the Sette and Buus laboratories and included the experimental data used to train NetMHC and NetCTL. The results obtained using this second dataset were very similar to those obtained using the first, independent dataset (figure S3 B).


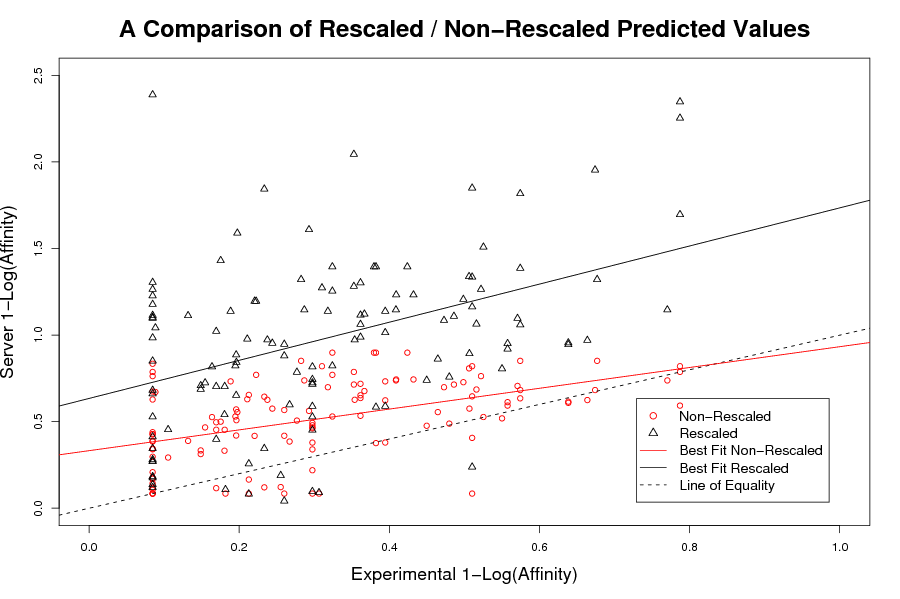


Figure S3 A: The experimental binding affinities for 128 epitopes were obtained from IEDB [1] and converted to a log scale (1 – Log_50000_ (nM Affinity)). These epitopes were then tested using NetMHC v3.0 to produce 2 sets of predicted binding affinities; rescaled or non-rescaled. The predicted scores were also converted to a log scale (1 – Log_50000_ (nM Affinity)) and the non-parametric Spearman’s ρ was used to calculate the correlation between experimental and predicted data.


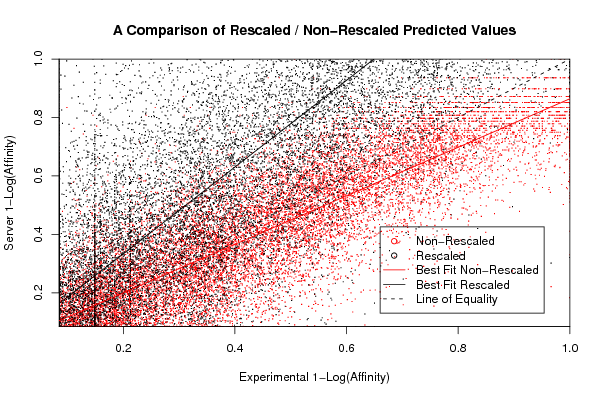


Figure S3 B: The experimental binding affinities for 29,336 epitopes were obtained from IEDB and converted to a log scale (1 – Log_50000_ (nM Affinity)). These epitopes were then tested using NetMHC v3.0 to produce 2 sets of predicted binding affinities; rescaled or non-rescaled. The predicted scores were also converted to a log scale (1 – Log_50000_ (nM Affinity)) and the non-parametric Spearman’s ρ was used to calculate the correlation between experimental and predicted data. The correlation between non-rescaled predicted affinities and experimental data showed a p-value of < 0.001 (Spearman’s ρ = 0.877). Rescaled predicted affinities and experimental data gave a p-value of < 0.001 (Spearman’s ρ = 0.816). The absolute difference between the 2 best-fit lines and the line of equality was calculated and it was shown that the non-rescaled values were significantly closer to the line of equality (Wilcoxon Paired Signed Rank Test; p-value < 0.001).

[1] Bjoern Peters, John Sidney, Phil Bourne, Huynh-Hoa Bui, Soeren Buus, Grace Doh, Ward Fleri, Mitch Kronenberg, Ralph Kubo, Ole Lund, David Nemazee, Julia V Ponomarenko, Muthu Sathiamurthy, Stephen Schoenberger, Scott Stewart, Pamela Surko, Scott Way, Steve Wilson, and Alessandro Sette. The immune epitope database and analysis resource: from vision to blueprint. *PLoS Biol*, 3(3):e91, Mar 2005.
