# Supplementary material for: T-Cell Epitope Prediction: Rescaling Can Mask Biological Variation between MHC Molecules
Source: PLoS Comput Biol. 2009 Mar 20;5(3):e1000327. doi: 10.1371/journal.pcbi.1000327 (PMC2650421; doi:10.1371/journal.pcbi.1000327)
Supplement: Figure S4 — A comparison of rescale values. (0.04 MB DOC) [file pcbi.1000327.s010.doc]

**Comparison of Rescale Values**

In the manuscript we calculated rescale values based on the predicted binding to 500,000 peptides selected at random from *Mycobacterium tuberculosis.* To check that the source of the peptides did not alter our conclusions we randomly selected 500,000 natural peptides from the Swiss-Prot database [1] and produced the top percentile re-scaling values for each allele from these peptides. Figure S5 compares these values to the re-scaling values we previously used, which were derived from non-overlapping peptides from *Mycobacterium tuberculosis.* As can be seen from figure S4 A, the two sets of rescale values are strongly positively correlated (R^2^=0.9563, p<0.001). Repeating our ROC curve analysis of rescaled and non-rescaled predictions from figure 1C using these new rescale factors (figure S4 B) gives very similar results to those reported in the manuscript. Consequently, whether we calculate the rescale factors using random natural peptides from *Mycobacterium tuberculosis* or on random natural peptides from a range of proteins, our conclusions remain unchanged.

**
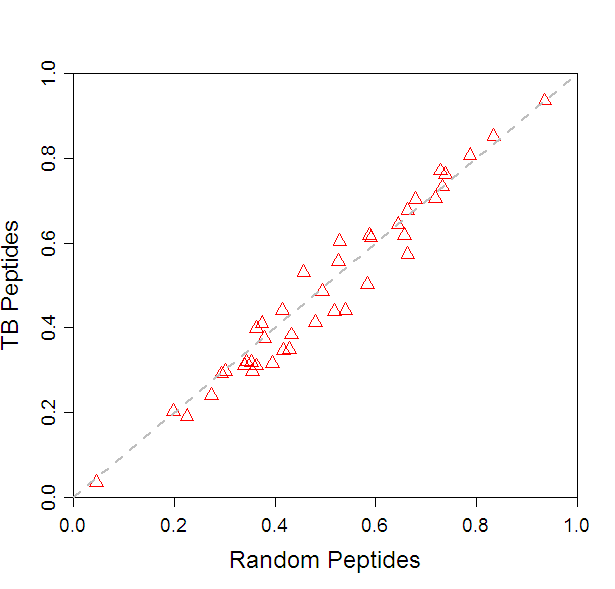
**

Figure S4 A: The relationship for the top percentile rescaling values for each allele between random natural peptides and non-overlapping peptides from *Mycobacterium tuberculosis.* There was no significant difference between the two measures (Mann-Whitney paired test, p=0.1181).


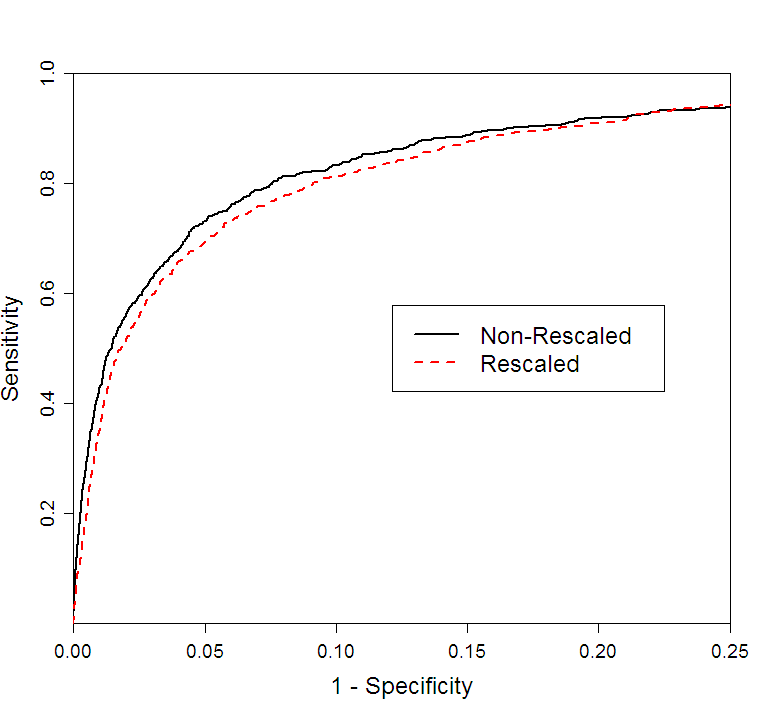


Figure S4 B: The ROC analysis of the Lanl^661^ dataset. The rescale values used are derived from random natural peptides, as opposed to peptides originating from *Mycobacterium tuberculosis.* The difference remains significant between the two curves (bootstrap test: p < 0.001).

[1] UniProt Consortium. The universal protein resource (UniProt). *Nucleic Acids Res*, 36(Database issue):D190–D195, Jan 2008.
